# Endoplasmic reticulum stress and quality control in relation to cisplatin resistance in tumor cells

**DOI:** 10.3389/fphar.2024.1419468

**Published:** 2024-06-14

**Authors:** Wentao Mu, Yao Zhi, Jianpeng Zhou, Chuanlei Wang, Kaiyuan Chai, Zhongqi Fan, Guoyue Lv

**Affiliations:** Department of Hepatobiliary and Pancreatic Surgery, General Surgery Center, First Hospital of Jilin University, Changchun, Jilin, China

**Keywords:** endoplasmic reticulum, cisplatin, chemoresistance, er stress, ERAD, ER-phagy

## Abstract

The endoplasmic reticulum (ER) is a crucial organelle that orchestrates key cellular functions like protein folding and lipid biosynthesis. However, it is highly sensitive to disturbances that lead to ER stress. In response, the unfolded protein response (UPR) activates to restore ER homeostasis, primarily through three sensors: IRE1, ATF6, and PERK. ERAD and autophagy are crucial in mitigating ER stress, yet their dysregulation can lead to the accumulation of misfolded proteins. Cisplatin, a commonly used chemotherapy drug, induces ER stress in tumor cells, activating complex signaling pathways. Resistance to cisplatin stems from reduced drug accumulation, activation of DNA repair, and anti-apoptotic mechanisms. Notably, cisplatin-induced ER stress can dualistically affect tumor cells, promoting either survival or apoptosis, depending on the context. ERAD is crucial for degrading misfolded proteins, whereas autophagy can protect cells from apoptosis or enhance ER stress-induced apoptosis. The complex interaction between ER stress, cisplatin resistance, ERAD, and autophagy opens new avenues for cancer treatment. Understanding these processes could lead to innovative strategies that overcome chemoresistance, potentially improving outcomes of cisplatin-based cancer treatments. This comprehensive review provides a multifaceted perspective on the complex mechanisms of ER stress, cisplatin resistance, and their implications in cancer therapy.

## 1 Introduction

The endoplasmic reticulum (ER) is a key organelle in cells, crucial for synthesizing, folding, and modifying secretory and transmembrane proteins (Oakes and Papa, 2015; Wang and Kaufman, 2016). Cells face various pressures that threaten their survival, including hypoxia and starvation. Facing these challenges, cells undergo ER stress, activating the unfolded protein response (UPR) (Urra et al., 2016; Cubillos-Ruiz et al., 2017). The UPR is an adaptive mechanism allowing cells to mitigate stress by restoring ER function and implementing quality control measures (Lebeaupin et al., 2018). Specifically, it involves recruiting ER proteins for proteasomal degradation through the endoplasmic reticulum-associated degradation (ERAD) mechanism (Chee et al., 2022; Xu et al., 2022). Additionally, macroautophagy, a lysosomal-mediated protein degradation pathway, plays a key role in recovering and clearing misfolded proteins, aggregated proteins, and damaged organelles, acting as a crucial protective mechanism during ER stress (Song et al., 2018). Furthermore, the ER autophagy pathway, with distinct regulatory mechanisms and responses, also governs this complex ER quality control process (Chino and Mizushima, 2020). External factors and internal events can disrupt this highly regulated process, leading to ER stress characterized by the accumulation of misfolded proteins.

During tumor development, genetic, transcriptional, and metabolic abnormalities occur due to inactivated tumor suppressor genes and oncogenic mutations. These abnormalities lead to an unfavorable microenvironment, inducing ER stress in tumor cells (Chen and Cubillos-Ruiz, 2021). It is crucial to recognize that tumor cells are heterogeneous, leading to varied responses and tolerance to ER stress. Therefore, sustained ER stress can affect tumor functionality and survival diversely, underlining its importance in understanding tumor biology (Lin et al., 2019). Cisplatin, a common chemotherapy drug, works by causing DNA damage and activating DNA damage response pathways, leading to cell apoptosis (Galluzzi et al., 2012; Ghosh, 2019; Li et al., 2023). Similarly, in tumors, cisplatin can induce ER stress in cells under treatment pressure (Linder and Shoshan, 2005; Gentilin et al., 2019; Zhu et al., 2023). However, cisplatin treatment often results in chemoresistance, making the treatment ineffective (Chen et al., 2020; Kryczka et al., 2021). Researchers are investigating the relationship between cisplatin-induced ER stress and chemotherapy resistance to understand contributing factors (Chen et al., 2020; Bahar et al., 2021).

This review aims to provide a comprehensive overview of the fundamental mechanisms of ER stress and its quality control. Elucidating the molecular mechanisms and signaling pathways involved offers insights into tumor cells’ adaptive responses and their impact on treatment outcomes. Understanding the interplay between ER stress and cisplatin resistance is crucial to advance tumor biology knowledge and develop personalized treatment strategies. Identifying novel therapeutic targets and designing more effective approaches can help overcome cisplatin resistance and enhance cancer treatment efficacy. In conclusion, this review explores the role of ER stress and its quality control mechanisms in cisplatin resistance in tumor cells. Exploring the molecular basis of cisplatin resistance and its link with ER stress aims to contribute to developing strategies to overcome chemoresistance and improve cisplatin-based cancer treatment outcomes.

## 2 ER stress and UPR activation: from molecular pathways to cellular outcomes

The ER, a critical organelle in eukaryotic cells, orchestrates key biological processes like Ca^2+^ homeostasis, protein folding and trafficking, and lipid biosynthesis (Zhang et al., 2014). Protein folding is particularly sensitive to environmental fluctuations. Nutrient deprivation, pathogenic stimuli, and hypoxia can cause misfolded proteins to accumulate in the ER, leading to cellular toxicity known as ER stress (Senft and Ronai, 2015; Hotamisligil and Davis, 2016). This stress response activates the UPR, which alleviates ER stress by removing misfolded proteins and restoring ER homeostasis (Zhang et al., 2014). Notably, ER stress plays a crucial role in many diseases, including metabolic disorders, neurological diseases, and cancer. This comprehensive review endeavors to provide an extensive overview of the current pathways and intricate molecular mechanisms that underlie ER stress and UPR activation.

Unfolded proteins in the ER lumen activate the UPR, driven by three sensors: Inositol-Requiring Enzyme 1 (IRE1), Activating Transcription Factor 6 (ATF6), and Protein kinase RNA-like ER Kinase (PERK). The highly expressed ER chaperone, Glucose-Regulated Protein 78 (GRP78, also HSPA5/Bip), binds to proteins’ hydrophobic domains, preventing misfolding during translocation to the ER (Oakes and Papa, 2015; Bhardwaj et al., 2020). Upon ER stress, when unfolded proteins reach a threshold and free GRP78 decreases, enough GRP78 dissociates from sensors, activating the downstream UPR pathway (Ibrahim et al., 2019). The activated UPR response restores ER homeostasis by regulating transcription and translation, enhances protein folding and turnover, and activates degradation pathways (Walter and Ron, 2011; Lemberg and Strisovsky, 2021). If these adaptive mechanisms fail to correct protein folding defects, cells undergo apoptosis, highlighting the ambivalent nature of ER stress (Hetz et al., 2020).

IRE1 consists of a serine/threonine protein kinase domain and an endoribonuclease domain, and is widely expressed in various tissues (Chen and Brandizzi, 2013). During ER stress, IRE1 releases GRP78 into the ER lumen, where it binds to unfolded proteins. Upon GRP78 dissociation, IRE1 undergoes dimerization and autophosphorylation, activating its endoribonuclease activity (Bhardwaj et al., 2020). The spliced form of X box-binding protein 1 (XBP1-S), produced after RNase cleavage, functions as a transcription factor that translocates to the nucleus (Qiu et al., 2023). This initiates the expression of genes coordinating protein transcription and translation, lipid synthesis, ERAD, and proteins maintaining ER homeostasis during stress. Additionally, IRE1-dependent decay (RIDD) reduces translation of secretion-related proteins during ER stress by degrading ER-located mRNA, relieving ER stress (Maurel et al., 2014). Conversely, activated IRE1 can form complexes with tumor necrosis factor-alpha receptor-associated factor 2 (TRAF2) and apoptosis signaling regulating kinase (ASK1) (Liu et al., 2020). TRAF2 activates Caspase12, which then activates Caspase9 and Caspase3, initiating apoptosis (Ge et al., 2015; Bhardwaj et al., 2020). Similarly, ASK1 activates pro-inflammatory and pro-apoptotic transcription factors like NF-κB, c-JUN, and AP-1 (El Jamal et al., 2016).

**FIGURE 1:**
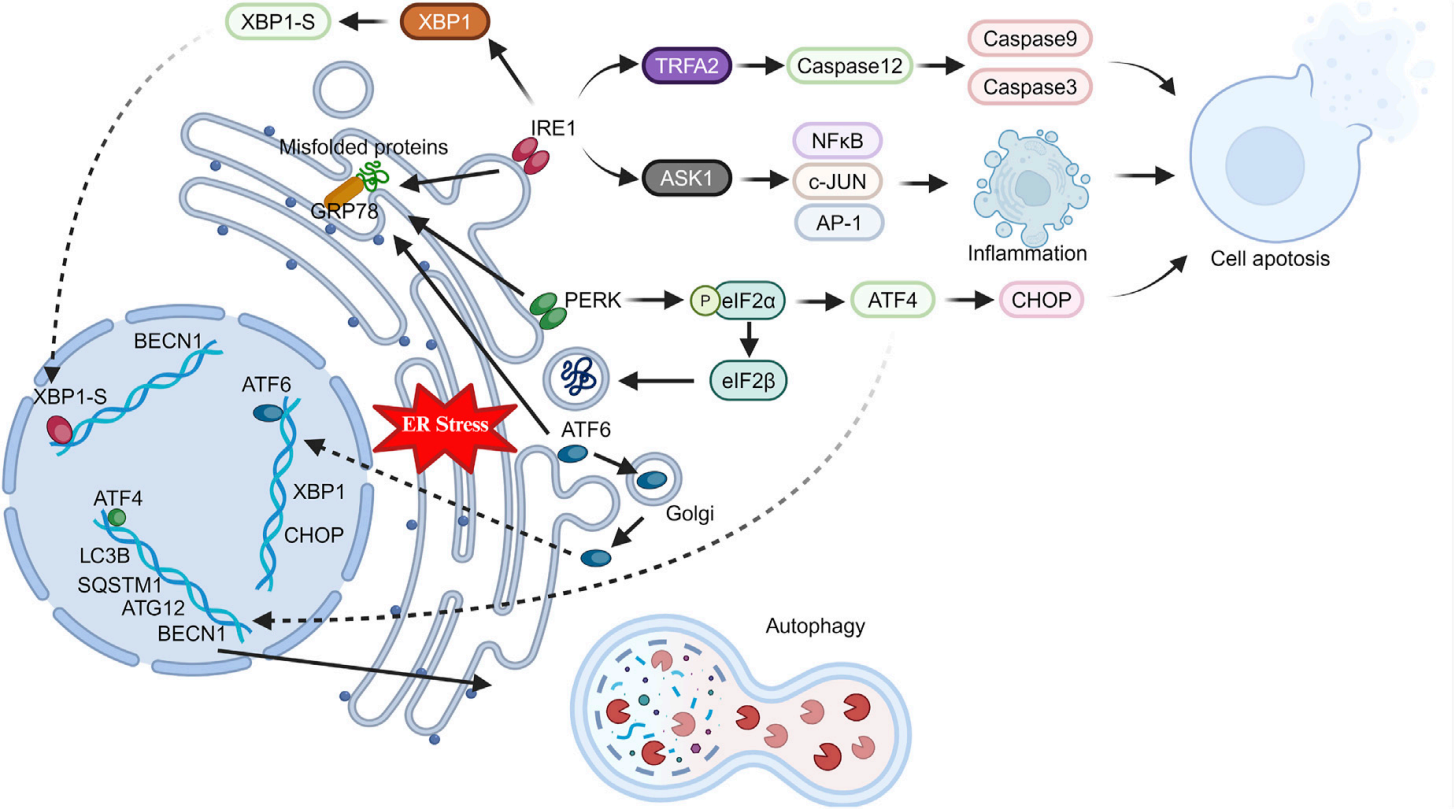
Changes in ER stress pathway-related proteins in stressed cells. Under stress, the endoplasmic reticulum triggers ER stress in cells. GRP78 activates key proteins (IRE1, PERK, ATF6), which in turn induce inflammation, apoptosis, and autophagy through intracellular and nuclear pathways.

After activation, ATF6 relocates to the Golgi apparatus, undergoing proteolysis to release its domain. Subsequently, like XBP1-S, the released ATF6 fragment functions as a transcription factor, translocating to the nucleus and upregulating ER stress-related genes (Bhardwaj et al., 2020; Ricciardi and Gnudi, 2020). PERK, another critical ER stress sensor like IRE1, is activated as a dimer through phosphorylation. Activated PERK primarily functions by phosphorylating eIF2α (Balsa et al., 2019). Phosphorylated eIF2α suppresses protein synthesis by inhibiting eIF2β, reducing translation and protein burden in the ER. Interestingly, phosphorylated eIF2α preferentially translates selective mRNA with an internal ribosomal entry site (IRES), like ATF4 (Hiraishi et al., 2014; Oakes and Papa, 2015; Bhardwaj et al., 2020). ATF4 upregulates pathways relieving ER stress and binds to promoters of autophagy-related genes like MAP1LC3B, ATG12, and BECN1, promoting their expression (Jiang et al., 2014; Wang et al., 2014; Tang et al., 2015). Additionally, ATF4 activates C/EBP homologous protein (CHOP) transcription, mediating cell death during ER stress (Rozpedek et al., 2016; Zielke et al., 2021). Therefore, activated PERK may shift cell fate towards adaptation or apoptosis, depending on ER stress severity and cell tolerability (Figure 1).

## 3 The machinery of endoplasmic reticulum-associated degradation (ERAD): substrate recognition, translocation, and ubiquitin-mediated degradation

The ERAD pathway is crucial for removing misfolded proteins from the ER (Römisch, 2005). It is a highly conserved surveillance system in eukaryotes, characterized by its coordinated organization. It involves substrate recognition, inverted translocation across the phospholipid bilayer, and transfer to the cytoplasm for degradation by the 26S proteasome after polyubiquitylation (Qi et al., 2017).

In the ERAD system, substrate recognition involves proteins with mutations, translation errors, or incorrect assembly (Nakatsukasa and Brodsky, 2008; Słomińska-Wojewódzka and Sandvig, 2015). Additionally, the abundance of specific ER proteins may be regulated in response to metabolic signals in the cell. Most proteins synthesized in the ER are modified by N-glycosylation of the mannose core oligosaccharide, crucial for ER quality control (Piirainen and Frey, 2022). This process’s products are deglycosylated by glucosidase I and II, allowing new glycoproteins to bind to calnexin (CNX) or calreticulin (CRT), facilitating oxidation, folding, and maturation. This also prevents deglycosylated glycoproteins from binding to CNX/CRT (Ruddock and Molinari, 2006; Clerc et al., 2009). Improperly folded proteins in the ER return to the CNX/CRT cycle for refolding through UGGT glycosylation (Doolittle et al., 2009). However, mutated or misfolded glycoproteins need to escape this cycle to enter ERAD. This process requires mannosidases to remove end mannose residues of the core oligosaccharide, facilitating interactions with mannosylated lectins before ERAD entry (Słomińska-Wojewódzka and Sandvig, 2015; Zhang et al., 2020). ER soluble proteins OS-9 and XTP3B/Erlectin recognize oligosaccharide-substrate proteins after deglycosylation or demannosylation via an MRH (Mannose 6-phosphate Receptor Homology) domain. Besides oligosaccharide-dependent ERAD, XTP3B and OS-9 are involved in non-glycosylated protein ERAD, relying on signals from mismatches in error-folding regions or glycosylation of mutated sites in non-glycosylated proteins (Kim et al., 2018). The XTP3B and OS-9 BiP complex might enable recognition of specific error-folding segments in non-glycosylated proteins, supporting non-glycosylated protein ERAD (Tang et al., 2014; van der Goot et al., 2018).

In ERAD, substrate recognition precedes the protein substrate’s transfer across the ER membrane into the cytosol for degradation by the ubiquitin-proteasome system (Zattas and Hochstrasser, 2015). Given most substrates are highly hydrophobic proteins, tight coordination between dislocation and degradation is essential to prevent cytosolic substrate aggregation (Cybulsky, 2013; Berner et al., 2018; Kang and Jeon, 2021). The dislocation complex, containing a ubiquitin ligase, integrates into the ER membrane, interacting with recognition factors like XTP3B or OS-9 by binding to SEL1L (Christianson et al., 2008). Once a substrate portion penetrates the ER membrane, cytosolic ATPase VCP/p97 facilitates its ATP-dependent unfolding and remodeling in most ERAD retrotranslocation processes (Sasset et al., 2015). VCP/p97 engages in various biological processes by recruiting cellular factors. Besides extracting substrates and altering conformations, VCP/p97’s role in proteasome-mediated degradation is linked to its UBX-binding domain (Ménager et al., 2014). This domain interacts with ubiquitin-binding proteins (Ufd1 and Npl4) and deubiquitinating enzymes (Yod1, VCIP135, Usp19, and Ataxin-3) to regulate protein degradation (Nowis et al., 2006; Erzurumlu et al., 2023).

The translocation complexes in ERAD for substrate recognition and degradation differ from those in protein import into the ER via the SEC61 translocon (Grotzke et al., 2017; Fregno and Molinari, 2019). The SEC61 translocon recognizes signal peptides for protein import and mediates transport of unfolded substrates through narrow transmembrane pores. In contrast, ERAD substrates have complex characteristics like folding and glycosylation modifications that SEC61 cannot accommodate (Hosomi et al., 2010). Evidence suggests the ERFAD and ERp90 complex can reduce disulfide bonds in SEL1L-associated glycoprotein substrates during translocation, possibly alleviating pore pressure (Riemer et al., 2011).

**FIGURE 2:**
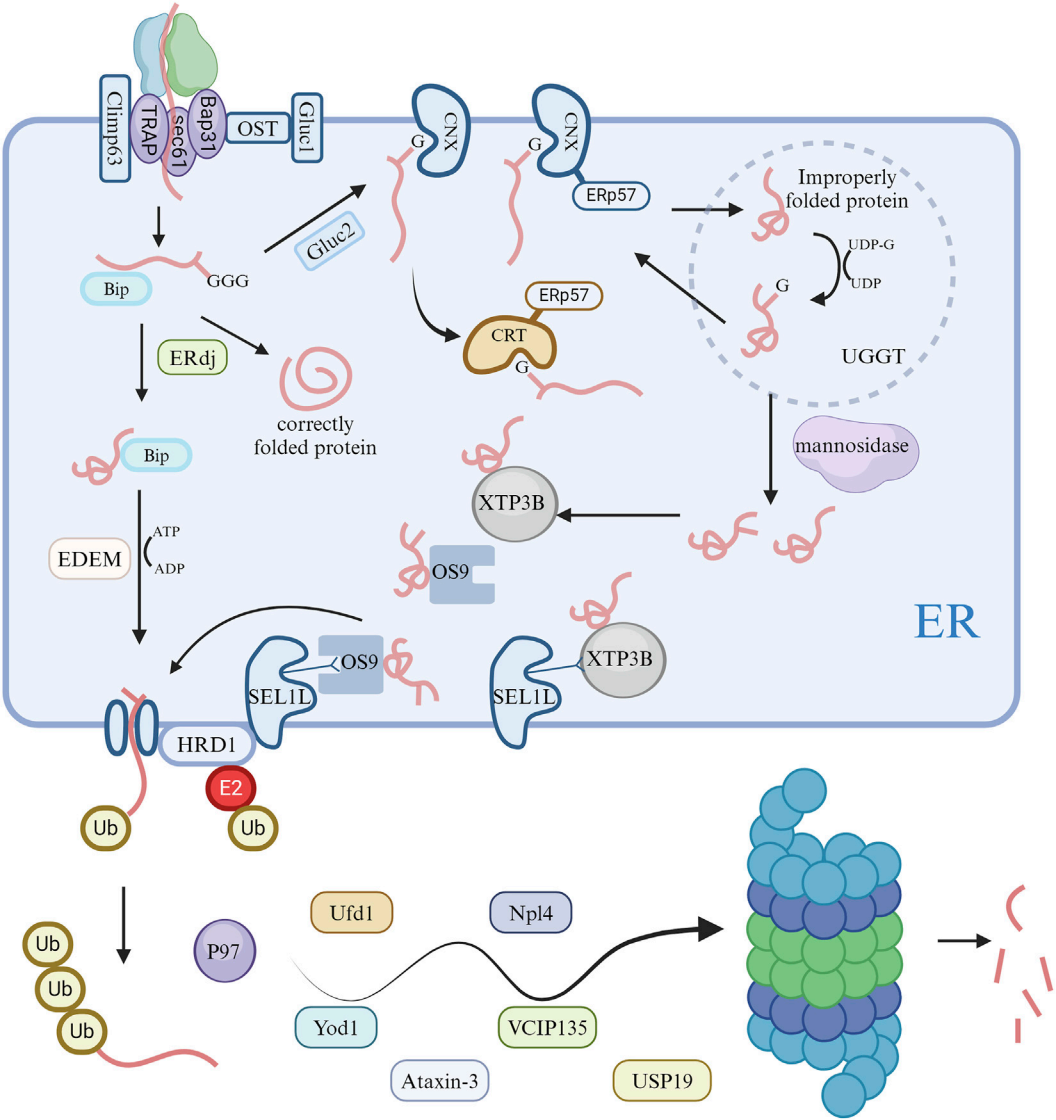
Activation of ERAD pathways and degradation of ubiquitin proteasomes in cells. Following unfolded protein responses in cells, the endoplasmic reticulum modifies proteins via the ERAD pathway, ultimately degrading misfolded proteins through the ubiquitin proteasome system.

The ubiquitination and subsequent degradation process involves enzyme-catalyzed, hierarchical reactions. Once activated by the E1 enzyme, E2 and E3 enzymes work together to attach ubiquitin to substrates for proteasome recognition (Fujita et al., 2007). Handling ERAD substrates properly requires multiple E3 ligases, involved in recognizing different ubiquitination sites on the substrate or coordinating and stabilizing its ubiquitination and deubiquitination. In mammalian ERAD pathways, essential E3 ubiquitin ligases include Hrd1/SYVN1 and gp78/AMFR, with others showing strong substrate-specific selectivity (Kim et al., 2015; Bhattacharya et al., 2018; Zhou et al., 2020) (Figure 2).

## 4 ER-phagy: dual mechanisms of Macro-ER-phagy and Micro-ER-phagy in cellular ER quality control

Besides ERAD, mediated by UPR, ER-phagy is a crucial component of ER quality control, offering complementary degradation mechanisms (Chino and Mizushima, 2020; Gubas and Dikic, 2022). Some ER-resident and few cytoplasmic proteins are degraded via the ER-phagy pathway, linking the ER domain to autophagic processes through receptors. ER-phagy can occur through autophagosomes (Macro-ER-phagy) or non-autophagosomes (Micro-ER-phagy) (Ferro-Novick et al., 2021). During Macro-ER-phagy, autophagosomes can non-selectively envelop cellular components, including organelles. However, studies on ER-phagy receptor functions reveal that Macro-ER-phagy can selectively target the ER.

ER-phagy adaptor proteins play a crucial role in ensuring selective autophagy. To date, six mammalian receptor proteins related to this process have been identified, including FAM134B, RTN3L, ATL3, SEC62, CCPG1, and TEX264 (Hübner and Dikic, 2020), all containing motifs to interact with LC3 or Atg8 (Ferro-Novick et al., 2021). Some receptors also interact with autophagy-initiating complexes like ULK1, ULK2, and FIP200 (Goodwin et al., 2017). Anchored to the ER, these proteins participate in Macro-ER-phagy by binding to LC3 via their LIR motif or to autophagy-initiating complexes (Hübner and Dikic, 2020).

Different receptor proteins serve various functions in ER-phagy. In mammals, FAM134B, RTN3L, ATL3, and TEX264 mediate starvation-induced ER-phagy, whereas SEC62 and CCPG1 are involved during ER stress and recovery (Preetha Rani et al., 2022). Receptor proteins with an RHD, like FAM134B and RTN3L, induce ER fragmentation through their short hairpin transmembrane domains, increasing membrane tension (DEletto et al., 2020). However, SEC62, CCPG1, and TEX264 lack the structural domains needed for such ER fragmentation. Instead, they cause molecular aggregation at ER-autophagic body contact sites, producing curvature and leading to membrane fragmentation. Additionally, the autophagic receptor P62 mediates ER-phagy by interacting with ubiquitin via the UBA domain and targeting autophagy through the LIR domain. The interaction between P62 and ubiquitinated TRIM13 has been reported to mediate ER-phagy. Micro-ER-phagy was first discovered in yeast. Under dithiothreitol-induced ER stress, ER whorls form and are specifically targeted by Micro-ER-phagy for vesicular transport and degradation (Reggiori and Molinari, 2022). Core autophagy factors like ULK1 and ULK2 are not needed in this process. Specific proteins like pre-collagen can be degraded via Micro-ER-phagy, differing from Macro-ER-phagy. However, the role of ATG family proteins in this process is still unclear (Ferro-Novick et al., 2021; Gubas and Dikic, 2022).

**FIGURE 3:**
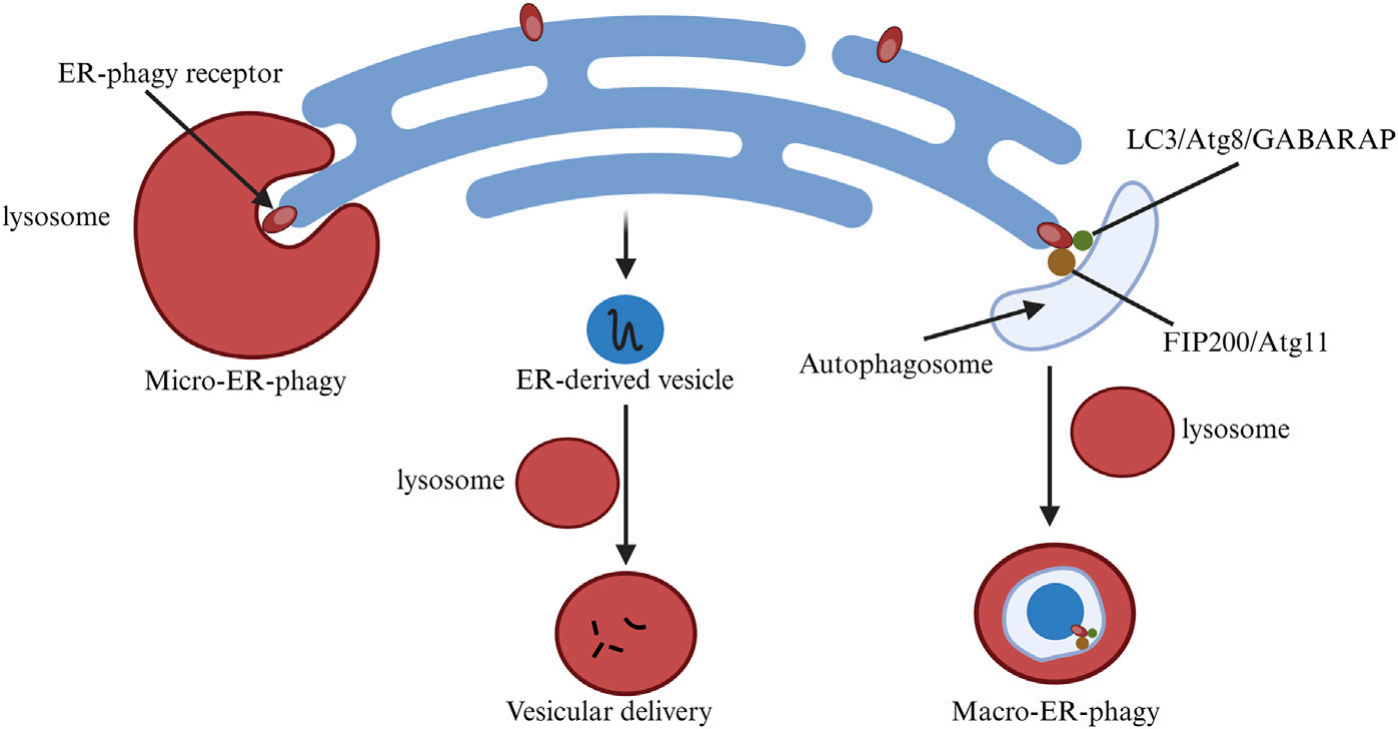
Changes in endoplasmic reticulum-phagy related pathways in cells. ER-phagy is classified into MacroER-phagy and MicroER-phagy, each degrading different protein types. The figure illustrates their differences.

As research into micro-autophagy progresses, it is found that micro-autophagy of endosomes rapidly degrades adaptor proteins like P62 and NDP52, relying on the ATG family binding system. This binding system might be linked to lysosomal membrane recognition, but its relationship with LC3, present on the ER in SEC62-mediated Micro-ER-phagy, remains uncertain (Gubas and Dikic, 2022). In summary, Micro-ER-phagy degrades targeted proteins within the ER without needing core autophagy factors such as ULK1 and ULK2. However, the role of ATG family proteins in this process is still unclear. Endosomal micro-autophagy degrades adaptor proteins via the ATG binding system, yet its relation to LC3 in ER-phagy needs further exploration (Figure 3).

## 5 Puzzle of cisplatin resistance in cancer therapy: mechanisms and strategies

Cisplatin is a common chemotherapy drug widely used for treating solid tumors (Ghosh, 2019; Romani, 2022). Despite its effectiveness against malignancies, the widespread use of cisplatin highlights the ongoing challenge of drug resistance in cancer therapy (Galluzzi et al., 2012; Galluzzi et al., 2014; Ashrafizadeh et al., 2020). Understanding the potential mechanisms of tumor resistance to cisplatin is crucial for developing new strategies to overcome this challenge.

**FIGURE 4:**
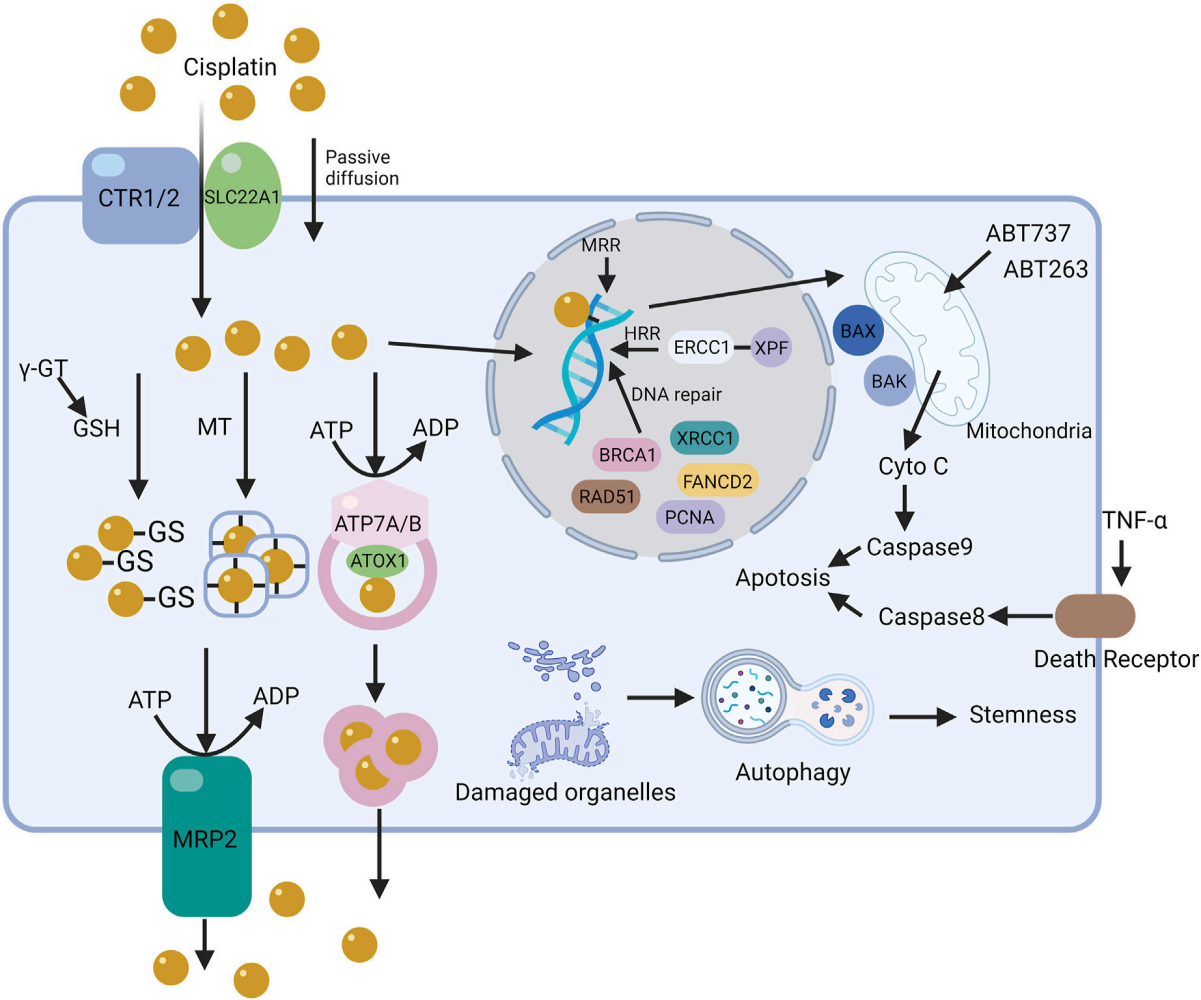
The mechanism of cisplatin resistance in cells. The resistance mechanisms to cisplatin in cells, based on its action pathways, are categorized into pre-target, on-target, and off-target resistance. The figure illustrates the core pathways of some resistance mechanisms in tumor cells.

Cisplatin resistance mainly stems from the reduced accumulation of cytotoxic compounds in cancer cells’ cytoplasm, protecting them from the activation of chemotherapy-induced DNA damage mechanisms (Dasari and Tchounwou, 2014). Like resistance development to other chemotherapeutic agents, cisplatin-resistant tumor cells gain additional genetic or epigenetic alterations, giving them a growth advantage like increased proliferative capacity (Ferreira et al., 2016). The cytotoxic effect of cisplatin involves a complex process from the drug’s entry into the cell to apoptosis induction, involving intricate mechanisms. Interfering with any stage of this process suppresses tumor cell apoptosis and leads to resistance. Resistance mechanisms arise from changes in intracellular molecules and pathways, leading to reduced interaction between cisplatin and DNA, interference with DNA damage signal activation, or both. Thus, cisplatin resistance involves various factors and is categorized into pre-target, on-target, post-target, and off-target resistance, based on mechanism of action (Galluzzi et al., 2012; Lugones et al., 2022) (Figure 4).

### 5.1 Pre-target

Pre-target resistance to cisplatin is linked to reduced cisplatin entry into tumor cells or increased cisplatin exclusion. The decrease in cisplatin accumulation is a key mechanism in the development of tumor cell resistance. Cisplatin entry into cells is influenced by sodium-potassium ion concentrations, pH, and the coordinated action of transport proteins and channels. Resistant tumor cells reduce copper and organic cation transporter protein expression on the cell membrane, lowering intracellular drug concentrations.

Copper transporter 1 (CTR1), widely expressed in tissues, is crucial for high-affinity copper uptake. CTR1 is thought to transport cisplatin, oxaliplatin, and carboplatin. Deleting the yeast CTR1 gene reduces cisplatin accumulation in cells and leads to resistance. Conversely, CTR1 overexpression makes small cell lung cancer cells sensitive to cisplatin, carboplatin, and oxaliplatin. The proteasome inhibitor bortezomib and the natural compound β-elemene prevent CTR1 degradation. However, contradictory conclusions have emerged about the impact of regulating CTR1 levels on sensitivity to platinum drugs. CRISPR-Cas9 genome editing to knock out CTR1, CTR2, ATOX1, and CCS had minimal impact on cisplatin sensitivity in HEK-293T and OVCAR8 cells. Another study showed that CTR1 overexpression did not increase platinum accumulation or affect cisplatin sensitivity in DLD-1 cells. The clinical relevance of CTR1 in platinum chemotherapy is also under question.

Copper transporter 2 (CTR2), a low-affinity copper transporter, is mainly found in late endosomes and lysosomes. The mRNA and protein levels of CTR2 significantly correlate with cisplatin sensitivity. CTR2 may affect cisplatin accumulation by influencing macropinocytosis, not by changing drug efflux or lysosomal storage. Additionally, CTR2 can interact with the CTR1 extracellular domain, stimulating CTR1 and reducing cisplatin accumulation in cells.

The Solute Carrier (SLC) superfamily includes over 300 members and 65 subfamilies, such as organic anion transport peptides, proteins, and organic cation transport proteins (OCTs). Cisplatin serves as a substrate for hOCT1 (SLC22A1), hOCT2 (SLC22A2), and hMATE1 (SLC47A1). Downregulation, mislocalization, or inhibited transport activity of OCTs can lower intracellular platinum levels. Omeprazole reportedly lowers OCT2 protein levels, leading to decreased cisplatin accumulation in cells. However, changes in SLC expression or distribution due to disease or drug interactions significantly affect the cellular uptake of therapeutic drugs, leading to suboptimal outcomes.

Early research on drug efflux processes and cell resistance was overlooked until studies on the multidrug resistance-associated protein (MRP) family revealed that several ABC membrane proteins are closely related to drug efflux from tumor cells. MRP2 plays a crucial role in cisplatin resistance, with an observed increase in MRP2 transporter proteins in resistant tumor cells.

Transport proteins like ATP7A and ATP7B, involved in cisplatin efflux, reduce cisplatin accumulation in tumor cells by enhancing functionality (Lukanović et al., 2020; Arnesano and Natile, 2021). After entering cells, cisplatin can bind to ATP7A/B’s CXXC motif, and this complex translocates to vesicles with Atox1 in an ATP-dependent manner. Tumor cells transfected with ATP7B show significantly increased resistance to cisplatin, suggesting downregulation of ATP7A/B as a potential method to overcome resistance.

Reduced glutathione (GSH) and metallothionein (MT) are crucial in cellular redox states, scavenging free radicals, protecting cells from external substances, and maintaining protein thiol groups. The active SH group of GSH has a high affinity for platinum, making it a simple, non-DNA-related target (Jamali et al., 2015; Sun et al., 2021; Luo et al., 2022). Upon entering the cytoplasm, GSH forms complexes with cisplatin, rendering it inactive in tumor cells. Lower intracellular chloride levels (<4 mmol/L) facilitate hydration reactions, allowing cisplatin to react with abundant GSH and metallothionein, reducing its accumulation in cells. Preventing resistance to the GSH pathway can be achieved with competitive inhibitors of GSH or by interfering with its synthesis. Gamma-glutamyltransferase (γ-GT), overexpressed in cisplatin-resistant tumor cells, catalyzes the production of cysteinylglycine from GSH, ten times more reactive with cisplatin than GSH itself. Excessive production of γ-GT may thus contribute to GSH-mediated cisplatin resistance in tumor cells. Similarly, MT’s richness in cysteine residues makes it a prime target for platinum chelation. Elevated MT levels are observed in both tumor tissues and the serum of cancer patients. RNA interference effectively inhibits MT overexpression, reversing platinum resistance. Thus, regardless of the pathway, reduced cisplatin accumulation in tumor cells leads to resistance.

### 5.2 On-target

DNA adduct formation is pivotal in the cytotoxicity of platinum-based antitumor drugs. Platinum-DNA complexes change the DNA structure, inhibiting replication, transcription, and inducing DNA double-strand breaks, thereby initiating repair mechanisms. If DNA repair fails or is overwhelmed by excessive damage, cell death follows. In on-target resistance, cells can survive cisplatin binding to DNA by activating repair mechanisms or tolerating genetic damage. In resistant tumor cells, accelerated recognition and repair of DNA adducts reduce apoptosis signal generation following DNA damage. Most intrastrand crosslinks are removed by the Nucleotide excision repair (NER) system, which excises damaged nucleotides and synthesizes DNA to restore genetic integrity; other damages are repaired through complex mechanisms.

NER plays a critical role in excising damaged nucleotides from DNA and restoring genetic integrity through DNA synthesis. ERCC1 and XPF proteins form the ERCC1-XPF complex, crucial for acting as an endonuclease in late-stage NER and homologous recombination repair (HRR) (Arora et al., 2010; Heyza et al., 2018). Additionally, increased tolerance to cisplatin is linked to the loss of function in the MMR pathway (Ray Chaudhuri et al., 2016). The MMR system corrects base errors occurring during DNA replication and recombination. During MMR, damage is detected through DNA intrastrand adduct recognition and apoptotic signal transmission. Downregulating key proteins MSH2 and MLH1 inhibits apoptosis in cisplatin-exposed tumor cells. NSCLC patients with high MSH2 expression after tumor resection have a better prognosis than those with low expression, reflecting differences in untreated tumors (Samimi et al., 2000; Ting et al., 2013). Tumors with high DNA repair capabilities may be less prone to recurrence in some settings but may also hinder the effectiveness of DNA damage-related chemotherapy. Cisplatin-induced interstrand adducts can cause double-strand breaks during the S phase or through DNA repair via homologous recombination. The HR system’s key proteins, BRCA1 and BRCA2, often mutated in breast and ovarian cancers, make tumors with HR defects more sensitive to platinum drugs. Like the MMR pathway, BRCA1-defective ovarian cancer, more prone to internal metastasis than sporadic cases, shows increased sensitivity to platinum drugs and better post-chemotherapy prognosis. The tumor’s DNA repair ability varies or even inversely correlates with malignancy and chemoresistance. Targeted therapy against these targets must be comprehensively and carefully considered to avoid diminished efficacy. Besides ERCC and BRCA, components like FANCD2, PCNA, XRCC1, and RAD51 play varied roles in DNA repair pathways. Understanding the DNA repair regulatory network will offer more accurate guidance for platinum-based chemotherapy.

### 5.3 Post-target

The development of resistance mechanisms to platinum-based drugs involves complex pathway alterations, especially in apoptotic signaling pathways triggered after DNA damage. Currently, two main apoptosis pathways are recognized: extrinsic and intrinsic. The extrinsic pathway activates when TNF family receptors bind to tumor necrosis factor receptors, initiating caspase-8 activation. The intrinsic pathway involves a balance shift between pro-apoptotic (e.g., BAX, BAK) and anti-apoptotic proteins (e.g., BCL-2, BCL-XL, BCL-w). Upon activation, pro-apoptotic signals lead to mitochondrial outer membrane permeabilization and cytochrome c release, triggering caspase cascade reactions.

Unrepaired DNA damage activates signals promoting apoptosis, linking gene and protein functionality to tumor resistance to platinum drugs. Platinum-resistant tumor cells have higher apoptosis thresholds, mainly from anti-apoptotic protein overexpression or mitochondrial signal transduction defects. Various factors, including survival signaling pathways, contribute to these adverse phenomena. TP53 gene inactivation prevents apoptosis induction via molecules like Bax, causing loss of apoptotic pathway activity and resistance in tumor cells. Ovarian cancer patients with wild-type TP53 genes respond better to platinum chemotherapy. Tetraploid tumor cells tolerate platinum drugs better than diploid cells, a phenomenon reversible by inhibiting or depleting TP53. Cisplatin induces apoptosis by activating MAPK1 and c-JUN N-terminal kinase, mediating FAS/FASL pathway signal transduction, unresponsive in resistant cells, reducing apoptosis and protecting tumor cells. The pro-apoptotic BCL2 family is closely linked to cisplatin sensitivity. Clinical trials are ongoing for combining BCL2 small molecule inhibitors (like ABT-263, ABT-737) with platinum drugs for tumor treatment. Caspase inactivation in apoptosis is also linked to tumor cisplatin resistance. Direct or indirect overactivation of pathways, such as the NFκB pathway, contributes to tumor cell resistance to platinum drugs (Xu J. et al., 2020; Abadi et al., 2022).

The tumor microenvironment (TME) plays a crucial role in cisplatin-resistant tumor cell survival, with mutual and dynamic interactions between TME and cancer cells. Evidence suggests that cancer-associated fibroblasts (CAFs) can induce tumor proliferation, metastasis, and chemotherapy resistance. CAFs secrete cytokines, proteins, or exosomal miRNAs activating anti-apoptotic pathways like PI3K/Akt, ANXA3/JNK, and IL-11/IL-11R/STAT3, providing resistance to tumor cells. CAFs also cause abnormal reshaping of extracellular substances, changes in tumor physical properties, or release cysteine and GSH, limiting intracellular platinum concentrations.

As mentioned earlier, the occurrence and development of ER stress in tumor cells can influence cell apoptosis, including the activation of Caspase family-related proteins, NF-κB and c-JUN related inflammatory pathways. In the context of cisplatin post-target resistance, ER quality control can regulate apoptosis signals. The ER links quality control-related proteins with cisplatin resistance by modulating apoptosis-related pathways. Thus, understanding ER quality control is crucial in studying cisplatin target resistance.

### 5.4 Off-target

Off-target resistance often involves changes in signaling pathways not directly related to cisplatin, yet influencing cisplatin-induced apoptosis. For instance, autophagy activation often induces cisplatin resistance, while inhibiting autophagy can restore tumor cell sensitivity to cisplatin, as observed in many tumor cell lines.

Autophagy, a “self-digestion” process, occurs in all eukaryotic organisms. It is essential for nutrient regulation, intracellular quality control, and homeostasis, recycling macromolecules as alternative energy sources in a defensive strategy. However, persistent or excessive autophagy can lead to cell death. Autophagy plays contradictory roles in tumor initiation and progression. It can promote either cell death or survival, depending on the cancer stage. After platinum therapy, an increase in drug-induced and basal autophagy is observed in platinum-resistant cells. Using agents such as 3-methyladenine or chloroquine to inhibit autophagy can enhance platinum-mediated cytotoxicity (Gąsiorkiewicz et al., 2021; Xu and Gewirtz, 2022).

Another aspect related to autophagy involves cancer stem cells (CSCs). CSCs, a subset of cancer cells in tumors, possess self-renewal and differentiation abilities. CSCs are considered significant contributors to drug resistance and recurrence. Autophagy regulates CSCs by protecting them and aiding in stem cell differentiation, somatic cell reprogramming, and self-renewal. Autophagy helps maintain stemness and chemotherapy resistance by regulating the expression of markers such as CD44, ABCB1, and ADAM17.

The autophagy process is closely related to the occurrence of ER stress and is a crucial component of ER quality control, contributing to off-target resistance to cisplatin. Consequently, the relationship between ER quality control and cisplatin resistance can be explored from the perspective of autophagy. This topic is currently a hot area of research in the field of drug resistance. A comprehensive understanding of ER quality control and cisplatin resistance will offer more precise insights and directions for effective drug resistance research.

## 6 Navigating cisplatin-induced ER stress: a dualistic pathway in tumor apoptosis and resistance

The ER is a crucial hub for regulating cellular homeostasis, vulnerable to various physiological and pathological changes that lead to ER stress. Recent findings show that tumor cells resistant to chemotherapy can also prevent ER stress-induced apoptosis (Haeri and Knox, 2012; Salaroglio et al., 2017). Given ER stress’s critical role in transmitting adaptive survival signals in cancer cells, it has become a focal point in chemotherapy resistance research. In normal cells, ATF6, IRE1, and PERK modulate ER stress (Kim et al., 2008); however, in cancer, oncogene activation and tumor suppressor loss lead to uncontrolled ER stress, aiding tumor cell survival under high protein synthesis and metabolic stress (Wang and Kaufman, 2014).

ER stress promotes cell survival by activating PERK, which phosphorylates eIF2α and Nrf2, causing cytosolic Nrf2 to dissociate from Keap1 (Cullinan et al., 2003; Del Vecchio et al., 2014). Nrf2 activation initiates the cytochrome P450 system and glutathione-S-transferases, reducing ROS and preventing tumor cell apoptosis under chemotherapy (Rodriguez-Antona and Ingelman-Sundberg, 2006; Lau et al., 2008). The PERK-Nrf2 pathway upregulates MRP1 through antioxidant enzymes (HO-1, SOD, catalase, *etc.*), neutralizing ROS and enhancing drug efflux (Healy et al., 2009). In chemotherapy-resistant HT29 cells, PERK upregulates MRP1 via Nrf2, protecting cells from chemotherapy by reducing ROS and increasing drug efflux, suggesting targeting this pathway could sensitize resistant cells to chemotherapy.

A strong positive correlation exists between Nrf2 levels and resistance to drugs like cisplatin, doxorubicin, and etoposide in various cancer cell lines (Ramos-Gomez et al., 2003). Stabilizing Nrf2 with antioxidative enzymes increases the Bcl-2/Bax ratio, reducing apoptosis and enhancing cell survival by modulating Topo II, p53, and p21 (Rotblat et al., 2012). Nrf2 is a potential cancer therapy target; for example, ML385, an Nrf2 inhibitor, selectively affects chemoresistance in NSCLC with Keap1 deficiency (Singh et al., 2016).

The Bcl-2 to Bax protein ratio significantly affects cell death and survival regulation. In H2S-treated mice, Nrf2 shows a protective effect by increasing Bcl-2 levels, protecting A549 cells from drug or radiation-induced DNA fragmentation and apoptosis (Calvert et al., 2009). After etoposide treatment, Bcl-2 overexpression is linked to increased apoptosis resistance. In both SCLC and NSCLC, etoposide treatment induces apoptosis and reduces Bcl-2 expression (Oizumi et al., 2002).

IRE1 recruits TRAF2 and activates ASK1, thus activating JNK and enhancing AP-1’s transcriptional activity, crucial for controlling apoptosis during stress. This increase in AP-1 DNA binding significantly boosts MCF-7 cell chemoresistance (Lewis-Wambi and Jordan, 2009). The role of ATF6α in creating chemoresistance via PDIA5 during ER stress has been identified, with PDIA5/ATF6α inhibition potentially restoring chemosensitivity (Morris et al., 1997).

Cisplatin can induce cell apoptosis through a non-nuclear DNA damage pathway. Research studies have demonstrated that cisplatin can elevate intracellular calcium levels and activate calcium protease-dependent Caspase 12, which in turn induces apoptosis in cells lacking a nucleus. Caspase 12 is closely associated with the ER (Mandic et al., 2003; Yu et al., 2007). Consequently, the ER might serve as an independent target for cisplatin-induced apoptosis by disrupting its protein folding function, thus inducing ER stress. Subsequent pathways, like the UPR or autophagy-related degradation, may play roles in preserving ER stability.

Inhibiting ER-specific Caspase 12 activity significantly reduces cisplatin-induced apoptosis, underscoring ER stress’s protective role. The upregulation of GRP78 after cisplatin treatment is noteworthy. Notably, this upregulation might not be closely related to ROS, as NAC (N-acetylcysteine) does not inhibit GRP78 upregulation or apoptosis after cisplatin treatment (Mandic et al., 2003; Yu et al., 2008). In various tumors, GRP78 acts as a protective protein, inhibiting apoptosis in response to cisplatin through different mechanisms. For example, in human melanoma cells, GRP78 reduces apoptosis by modulating the Caspase 4/Caspase 8 pathway. In breast and endometrial cancer, GRP78 enhances tumor cell resistance to cisplatin via the PARP1/Caspase 3/JNK pathway. In colorectal cancer, GRP78 lowers the apoptotic rate under cisplatin treatment via the Xbox1 pathway. Similarly, in HNSCC, nasopharyngeal carcinoma, and non-small-cell lung cancer, GRP78 mediates a reduction in apoptosis rate upon cisplatin exposure.

However, the compensatory capacity of ER stress is limited. When exposed to excessive detrimental stimuli, cells can undergo apoptosis due to overactivated ER stress, primarily mediated by CHOP. Cisplatin can induce excessive ER stress, upregulating the PERK/eIF2α/ATF4 pathway and promoting CHOP expression, thereby facilitating tumor cell apoptosis. This pathway has been confirmed in various tumor types, including non-small cell lung cancer (Tang et al., 2022), malignant pleural mesothelioma (Xu et al., 2018), neuroblastoma (Chen et al., 2013), and head and neck squamous-cell carcinoma (Mosca et al., 2019), among others. Inhibiting autophagy with 3-MA or CQ, or knocking out P62, intensifies cisplatin-induced ER stress, increasing the apoptotic rate of tumor cells during cisplatin treatment. Therefore, deficiency or impairment in UPR and autophagy can lead to excessive ER stress-induced apoptosis in cells treated with cisplatin.

Regarding cisplatin resistance, various tumor cells show either survival or apoptosis induction via the ER-stress pathway. The outcomes and pathway activations triggered by varying levels of ER stress are not uniform, highlighting ER stress’s complex and dual nature. Therefore, when developing anti-tumor therapies targeting ER stress, considering its dual effects and intricacies is vital. This is necessary to ensure the drugs’ functionality is not compromised or excessively altered.

## 7 Complex role of UPR in tumor microenvironment: from chemoresistance to apoptosis modulation

Certain tumor cells can facilitate tumor growth and chemoresistance in a stressful microenvironment by activating the UPR. UPR signaling molecules interact with oncogenes and tumor suppressor gene networks, regulating their functions in cancer development. Understanding the specific molecular mechanisms involved is crucial to comprehend UPR’s role. It is important to note that excessive ER stress-induced UPR can lead to cell death, introducing complexity and duality in designing drugs targeting UPR.

UPR plays a protective role in drug resistance, especially in tumor cells with enhanced cisplatin resistance. In hepatocellular carcinoma, UPR inhibits cisplatin-induced apoptosis, potentially involving Hsp27. Hsp27 activation can suppress cell death in HCC cells by promoting autophagy (Chen et al., 2011). Similarly, GRP78-mediated UPR reduces cisplatin-induced apoptosis in hypopharyngeal carcinoma (Pi et al., 2014) and glioblastomas (Hussein et al., 2022), promoting tumor cell survival. In pancreatic cancer, UPR mitigates cisplatin-induced cell death through ER dilation and intracellular Ca^2+^ modulation, which Bortezomib can inhibit (Nawrocki et al., 2005). UPR can directly or indirectly attenuate apoptosis in response to cisplatin chemotherapy in certain tumor types. For example, in osteosarcoma, UPR suppresses apoptosis by modulating the NF-κB pathway (Yan et al., 2015). In malignant melanoma, EF24-mediated UPR inhibits apoptosis (He et al., 2021). Similarly, in hypopharyngeal carcinoma, hypoxia-induced UPR reduces the apoptosis rate (Pi et al., 2014).

In the KB-3 human epidermoid carcinoma cell line, cisplatin resistance may link to mutations in the ERAD system. It is proposed that cisplatin-resistant cells show elevated levels of M8.1, a high-mannose-type glycan (Nakagawa et al., 2008). Moreover, UPR can enhance the cytotoxic effects of cisplatin in certain tumors, mainly through ER stress-mediated mechanisms. In malignant pleural mesothelioma (Xu et al., 2018) and mesothelioma (Zhang et al., 2010), UPR induces apoptosis in response to cisplatin via the PERK/eIF2α/ATF4 and GRP78 pathways. In ovarian carcinoma cells, ONC201-triggered UPR promotes apoptosis through the ER stress pathway involving ATF3 and ATF4. Furthermore, UPR can directly mediate apoptosis. In lung cancer, UPR induces apoptosis by engaging caspase 2 and DNA repair mechanisms, with HSF1’s participation (Gaddameedhi and Chatterjee, 2009; Zhang et al., 2023).

## 8 Autophagy in chemotherapy resistance: dual role in survival and sensitization of cancer cells

Autophagy, crucial for degrading and recycling cellular components, has emerged as a key contributor to chemotherapy resistance (Ishaq et al., 2020). Although primarily maintaining cellular homeostasis and promoting survival during stress, autophagy’s role in chemotherapy-treated cancer cells is complex (Smith and Macleod, 2019; Zamame Ramirez et al., 2021). In some cases, autophagy acts as a protective mechanism, enabling cancer cells to adapt to chemotherapy-induced stress and fostering resistance (Sui et al., 2013; Ferreira et al., 2021). By eliminating damaged cellular components and providing energy, autophagy can support cancer cell survival even with cytotoxic drugs present. Cisplatin therapy often promotes autophagy, with the link to cisplatin resistance identified as early as 2010. In lung adenocarcinoma A549, cisplatin-resistant cell lines show increased autophagy (Ren et al., 2010). Autophagy inhibition is common in cisplatin-sensitive cells, whereas it is activated in cisplatin-resistant tumor cells. Thus, inhibiting autophagy has become an effective strategy to reduce cisplatin resistance in tumor cells.

Autophagy activation occurs in both cisplatin-sensitive and -resistant cells after treatment. Thus, cisplatin resistance may not solely result from autophagy. As is well known, autophagy can participate in cell survival (protective autophagy) and cell death (cytotoxic autophagy) (Yu and Klionsky, 2022). Detecting the type of autophagy induced after cisplatin treatment is crucial, but currently, no reliable method exists to distinguish between autophagy types. Therefore, cisplatin induced autophagy can participate in both the promotion of tumor cell survival and the killing of tumor cells. However, the link between autophagy and chemotherapy resistance is not universally consistent (Huang et al., 2018; Xu Z. et al., 2020; Zhang and Liu, 2021). In certain scenarios, excessive autophagy may lead to cell death. Researchers are exploring the molecular mechanisms behind autophagy’s dual role and strategies to modulate its activity to enhance chemotherapy sensitivity in cancer cells. Understanding how autophagy contributes to chemotherapy resistance is crucial for developing more effective treatments against resistant cancers.

In addition, Cisplatin resistance may relate to changes in mitophagy. Cisplatin activates DRP1, leading to mitochondrial division. Dysfunctional mitochondria are removed via mitophagy, a process linked to cisplatin resistance. Mdivi-1 enhanced cisplatin sensitivity in hepatocellular carcinoma by inhibiting mitophagy via DRP1 (Ma et al., 2020). In cisplatin-resistant ovarian cancer, mitochondria become more fragmented, and BNIP3 expression increases. Silencing BNIP3 increases cisplatin sensitivity in drug-resistant cells. Mitochondrial morphology and function are closely related to cisplatin resistance (Vianello et al., 2022). While various factors influence mitochondria, mitophagy plays a significant role.

Although no current research proves the relationship between ER-phagy and cisplatin resistance, increasing studies on ER-phagy junction proteins highlight ER-phagy’s significant role in chemotherapy resistance. For instance, METTL3-mediated m6A modification upregulates the ER-phagy protein Sec62 via β-Catenin, enhancing Wnt signaling and promoting stemness and chemotherapy resistance in colorectal cancer (Liu et al., 2021). Additionally, recent studies link FAM134B mutations to tumor drug resistance, with evidence showing that lysosomal degradation of FAM134B contributes to tumor cell survival (Chipurupalli et al., 2022). With growing scientific interest in ER-phagy, further research is expected to unveil its correlation with chemotherapy resistance.

## 9 Discussion

The ER is crucial in coordinating cellular functions like protein folding, transportation, and modification (Oakes and Papa, 2015; Urra et al., 2016). The following discussion provides a comprehensive view on these interconnected processes and their implications for developing effective cancer treatment strategies. Cisplatin, a key chemotherapy agent, has a dual nature. While effectively triggering DNA damage responses and apoptosis, prolonged use often leads to chemoresistance (Gentilin et al., 2019). Delineating cisplatin resistance categories from pre-target to off-target offers avenues for tailored therapeutic approaches against resistance. However, the novel perspective lies in the link between cisplatin and ER stress. Once cisplatin enters tumor cells, activated ER-stress serves both as a sword to destroy tumor cells and a shield against cisplatin’s lethal effects. This dual role of ER-stress exemplifies its complex nature in chemotherapy resistance. Consequently, researching ER-stress is considered a challenging yet intriguing aspect of studying chemotherapy resistance. Combining cisplatin-induced ER stress with chemotherapy resistance introduces a new dimension to resistance, emphasizing the need for ER stress modulation in therapy strategies.

This review explores the complex mechanics of ERAD and its interplay with ER stress and cisplatin resistance. ERAD’s role in reducing ER stress through targeted protein degradation, along with its protective function in tumor cells, provides insight into tumor adaptation. The understanding that ERAD and ER stress direct tumor cell responses to cisplatin reaffirms their crucial role in driving cancer progression and resistance. The ERAD pathway, crucial for clearing misfolded proteins in the endoplasmic reticulum, significantly contributes to maintaining intracellular protein homeostasis. The capacity to synthesize and utilize basic proteins allows cells to enter a dormant or low-consumption survival state under the intense stress of chemotherapy. This cellular state can gradually revert post-chemotherapy, potentially leading to the failure of tumor treatment. Thus, regulating the ERAD process in tumor cells during chemotherapy is crucial for the treatment’s success or failure.

Autophagy emerges as a significant yet complex factor in chemotherapy resistance. Its dual role in maintaining tumor cell survival during stress and potentially driving apoptosis creates a nuanced balance. Understanding the complex relationship between autophagy and chemotherapy resistance opens avenues to target autophagy therapeutically, to either enhance resistance or sensitize cells to chemotherapy stress. Particularly, the section on ER-phagy in our paper is anticipated to become a focal area in future chemotherapy resistance research. Firstly, autophagy influences this mechanism’s functional regulation, indirectly maintaining endoplasmic reticulum homeostasis. The combined use of drugs in this process may yield unexpected benefits. Additionally, varying ER-phagy capabilities across tumor cells might explain the differential responses to autophagy inhibitors. Therefore, it is crucial to assess and understand endoplasmic reticulum homeostasis-related pathways while regulating autophagy to prevent counterproductive effects.

Looking forward, a holistic understanding of ER stress, cisplatin resistance, and their interplay with ERAD and autophagy guides us towards innovative treatment paradigms. Developing strategies to modulate ER stress responses, utilize ERAD machinery, and leverage autophagy’s dual nature could overcome chemotherapy resistance and improve treatment efficacy. By understanding these pathways, we can swiftly identify chemotherapy-resistant tumors’ resistance mechanisms and target them with specific medications. The complex aspects of these processes call for future research to uncover finer details, paving the way for personalized therapies that could transform cancer treatment outcomes.

## References

[B1] Abadi A. J. Mirzaei S. Mahabady M. K. Hashemi F. Zabolian A. Hashemi F. (2022). Curcumin and its derivatives in cancer therapy: potentiating antitumor activity of cisplatin and reducing side effects. Phytother. Res. 36, 189–213. 10.1002/ptr.7305 34697839

[B2] Arnesano F. Natile G. (2021). Interference between copper transport systems and platinum drugs. Semin. Cancer Biol. 76, 173–188. 10.1016/j.semcancer.2021.05.023 34058339

[B3] Arora S. Kothandapani A. Tillison K. Kalman-Maltese V. Patrick S. M. (2010). Downregulation of XPF-ERCC1 enhances cisplatin efficacy in cancer cells. DNA Repair (Amst) 9, 745–753. 10.1016/j.dnarep.2010.03.010 20418188 PMC4331052

[B4] Ashrafizadeh M. Zarrabi A. Hushmandi K. Kalantari M. Mohammadinejad R. Javaheri T. (2020). Association of the epithelial-mesenchymal transition (EMT) with cisplatin resistance. Int. J. Mol. Sci. 21, 4002. 10.3390/ijms21114002 32503307 PMC7312011

[B5] Bahar E. Kim J. Y. Kim D. C. Kim H. S. Yoon H. (2021). Combination of niraparib, cisplatin and twist knockdown in cisplatin-resistant ovarian cancer cells potentially enhances synthetic lethality through ER-stress mediated mitochondrial apoptosis pathway. Int. J. Mol. Sci. 22, 3916. 10.3390/ijms22083916 33920140 PMC8070209

[B6] Balsa E. Soustek M. S. Thomas A. Cogliati S. García-Poyatos C. Martín-García E. (2019). ER and nutrient stress promote assembly of respiratory chain supercomplexes through the PERK-eIF2α Axis. Mol. Cell 74, 877–890. 10.1016/j.molcel.2019.03.031 31023583 PMC6555668

[B7] Berner N. Reutter K. R. Wolf D. H. (2018). Protein quality control of the endoplasmic reticulum and ubiquitin-proteasome-triggered degradation of aberrant proteins: yeast pioneers the path. Annu. Rev. Biochem. 87, 751–782. 10.1146/annurev-biochem-062917-012749 29394096

[B8] Bhardwaj M. Leli N. M. Koumenis C. Amaravadi R. K. (2020). Regulation of autophagy by canonical and non-canonical ER stress responses. Semin. Cancer Biol. 66, 116–128. 10.1016/j.semcancer.2019.11.007 31838023 PMC7325862

[B9] Bhattacharya A. Sun S. Wang H. Liu M. Long Q. Yin L. (2018). Hepatic Sel1L-Hrd1 ER-associated degradation (ERAD) manages FGF21 levels and systemic metabolism via CREBH. Embo J. 37, e99277. 10.15252/embj.201899277 30389665 PMC6236331

[B10] Calvert J. W. Jha S. Gundewar S. Elrod J. W. Ramachandran A. Pattillo C. B. (2009). Hydrogen sulfide mediates cardioprotection through Nrf2 signaling. Circ. Res. 105, 365–374. 10.1161/circresaha.109.199919 19608979 PMC2735849

[B11] Chee Y. H. Samali A. Robinson C. M. (2022). Unfolded protein response at the cross roads of tumourigenesis, oxygen sensing and drug resistance in clear cell renal cell carcinoma. Biochim. Biophys. Acta Rev. Cancer 1877, 188814. 10.1016/j.bbcan.2022.188814 36195277

[B12] Chen C. Y. Kawasumi M. Lan T. Y. Poon C. L. Lin Y. S. Wu P. J. (2020). Adaptation to endoplasmic reticulum stress enhances resistance of oral cancer cells to cisplatin by up-regulating polymerase η and increasing DNA repair efficiency. Int. J. Mol. Sci. 22, 355. 10.3390/ijms22010355 33396303 PMC7794796

[B13] Chen R. Dai R. Y. Duan C. Y. Liu Y. P. Chen S. K. Yan D. M. (2011). Unfolded protein response suppresses cisplatin-induced apoptosis via autophagy regulation in human hepatocellular carcinoma cells. Folia Biol. (Praha) 57, 87–95. 21888831 10.14712/fb2011057030087

[B14] Chen X. Cubillos-Ruiz J. R. (2021). Endoplasmic reticulum stress signals in the tumour and its microenvironment. Nat. Rev. Cancer 21, 71–88. 10.1038/s41568-020-00312-2 33214692 PMC7927882

[B15] Chen Y. Brandizzi F. (2013). IRE1: ER stress sensor and cell fate executor. Trends Cell Biol. 23, 547–555. 10.1016/j.tcb.2013.06.005 23880584 PMC3818365

[B16] Chen Y. Tsai Y. H. Tseng S. H. (2013). RECK regulated endoplasmic reticulum stress response and enhanced cisplatin-induced cell death in neuroblastoma cells. Surgery 154, 968–979. 10.1016/j.surg.2013.05.026 24084596

[B17] Chino H. Mizushima N. (2020). ER-phagy: quality control and turnover of endoplasmic reticulum. Trends Cell Biol. 30, 384–398. 10.1016/j.tcb.2020.02.001 32302550

[B18] Chipurupalli S. Desiderio V. Robinson N. (2022). Analysis of ER-phagy in cancer drug resistance. Methods Mol. Biol. 2535, 211–220. 10.1007/978-1-0716-2513-2_16 35867233

[B19] Christianson J. C. Shaler T. A. Tyler R. E. Kopito R. R. (2008). OS-9 and GRP94 deliver mutant alpha1-antitrypsin to the Hrd1-SEL1L ubiquitin ligase complex for ERAD. Nat. Cell Biol. 10, 272–282. 10.1038/ncb1689 18264092 PMC2757077

[B20] Clerc S. Hirsch C. Oggier D. M. Deprez P. Jakob C. Sommer T. (2009). Htm1 protein generates the N-glycan signal for glycoprotein degradation in the endoplasmic reticulum. J. Cell Biol. 184, 159–172. 10.1083/jcb.200809198 19124653 PMC2615083

[B21] Cubillos-Ruiz J. R. Bettigole S. E. Glimcher L. H. (2017). Tumorigenic and immunosuppressive effects of endoplasmic reticulum stress in cancer. Cell 168, 692–706. 10.1016/j.cell.2016.12.004 28187289 PMC5333759

[B22] Cullinan S. B. Zhang D. Hannink M. Arvisais E. Kaufman R. J. Diehl J. A. (2003). Nrf2 is a direct PERK substrate and effector of PERK-dependent cell survival. Mol. Cell Biol. 23, 7198–7209. 10.1128/mcb.23.20.7198-7209.2003 14517290 PMC230321

[B23] Cybulsky A. V. (2013). The intersecting roles of endoplasmic reticulum stress, ubiquitin-proteasome system, and autophagy in the pathogenesis of proteinuric kidney disease. Kidney Int. 84, 25–33. 10.1038/ki.2012.390 23254900

[B24] Dasari S. Tchounwou P. B. (2014). Cisplatin in cancer therapy: molecular mechanisms of action. Eur. J. Pharmacol. 740, 364–378. 10.1016/j.ejphar.2014.07.025 25058905 PMC4146684

[B25] DEletto M. Oliverio S. Di Sano F. (2020). Reticulon Homology domain-containing proteins and ER-phagy. Front. Cell Dev. Biol. 8, 90. 10.3389/fcell.2020.00090 32154249 PMC7047209

[B26] Del Vecchio C. A. Feng Y. Sokol E. S. Tillman E. J. Sanduja S. Reinhardt F. (2014). De-differentiation confers multidrug resistance via noncanonical PERK-Nrf2 signaling. PLoS Biol. 12, e1001945. 10.1371/journal.pbio.1001945 25203443 PMC4159113

[B27] Doolittle M. H. Ben-Zeev O. Bassilian S. Whitelegge J. P. Péterfy M. Wong H. (2009). Hepatic lipase maturation: a partial proteome of interacting factors. J. Lipid Res. 50, 1173–1184. 10.1194/jlr.M800603-JLR200 19136429 PMC2681399

[B28] El Jamal S. M. Taylor E. B. Abd Elmageed Z. Y. Alamodi A. A. Selimovic D. Alkhateeb A. (2016). Interferon gamma-induced apoptosis of head and neck squamous cell carcinoma is connected to indoleamine-2,3-dioxygenase via mitochondrial and ER stress-associated pathways. Cell Div. 11, 11. 10.1186/s13008-016-0023-4 27486476 PMC4969639

[B29] Erzurumlu Y. Catakli D. Dogan H. K. (2023). Circadian oscillation pattern of endoplasmic reticulum quality control (ERQC) components in human embryonic kidney HEK293 cells. J. Circadian Rhythms 21 (1), 1. 10.5334/jcr.219 37033333 PMC10077977

[B30] Ferreira J. A. Peixoto A. Neves M. Gaiteiro C. Reis C. A. Assaraf Y. G. (2016). Mechanisms of cisplatin resistance and targeting of cancer stem cells: adding glycosylation to the equation. Drug Resist Updat 24, 34–54. 10.1016/j.drup.2015.11.003 26830314

[B31] Ferreira P. M. P. Sousa R. W. R. Ferreira J. R. O. Militão G. C. G. Bezerra D. P. (2021). Chloroquine and hydroxychloroquine in antitumor therapies based on autophagy-related mechanisms. Pharmacol. Res. 168, 105582. 10.1016/j.phrs.2021.105582 33775862

[B32] Ferro-Novick S. Reggiori F. Er-Phagy J. L. Homeostasis E. R. (2021). ER-phagy, ER homeostasis, and ER quality control: implications for disease. Trends Biochem. Sci. 46, 630–639. 10.1016/j.tibs.2020.12.013 33509650 PMC8286283

[B33] Fregno I. Molinari M. (2019). Proteasomal and lysosomal clearance of faulty secretory proteins: ER-associated degradation (ERAD) and ER-to-lysosome-associated degradation (ERLAD) pathways. Crit. Rev. Biochem. Mol. Biol. 54, 153–163. 10.1080/10409238.2019.1610351 31084437

[B34] Fujita E. Kouroku Y. Isoai A. Kumagai H. Misutani A. Matsuda C. (2007). Two endoplasmic reticulum-associated degradation (ERAD) systems for the novel variant of the mutant dysferlin: ubiquitin/proteasome ERAD(I) and autophagy/lysosome ERAD(II). Hum. Mol. Genet. 16, 618–629. 10.1093/hmg/ddm002 17331981

[B35] Gaddameedhi S. Chatterjee S. (2009). Association between the unfolded protein response, induced by 2-deoxyglucose, and hypersensitivity to cisplatin: a mechanistic study employing molecular genomics. J. Cancer Res. Ther. 5 (Suppl. 1), S61–S66. 10.4103/0973-1482.55146 20009298

[B36] Galluzzi L. Senovilla L. Vitale I. Michels J. Martins I. Kepp O. (2012). Molecular mechanisms of cisplatin resistance. Oncogene 31, 1869–1883. 10.1038/onc.2011.384 21892204

[B37] Galluzzi L. Vitale I. Michels J. Brenner C. Szabadkai G. Harel-Bellan A. (2014). Systems biology of cisplatin resistance: past, present and future. Cell Death Dis. 5, e1257. 10.1038/cddis.2013.428 24874729 PMC4047912

[B38] Gąsiorkiewicz B. M. Koczurkiewicz-Adamczyk P. Piska K. Pękala E. (2021). Autophagy modulating agents as chemosensitizers for cisplatin therapy in cancer. Invest. New Drugs 39, 538–563. 10.1007/s10637-020-01032-y 33159673 PMC7960624

[B39] Ge W. Yin Q. Xian H. (2015). Wogonin induced mitochondrial dysfunction and endoplasmic reticulum stress in human malignant neuroblastoma cells via ire1α-dependent pathway. J. Mol. Neurosci. 56, 652–662. 10.1007/s12031-015-0530-9 25740014

[B40] Gentilin E. Simoni E. Candito M. Cazzador D. Astolfi L. (2019). Cisplatin-induced ototoxicity: updates on molecular targets. Trends Mol. Med. 25, 1123–1132. 10.1016/j.molmed.2019.08.002 31473143

[B41] Ghosh S. (2019). Cisplatin: the first metal based anticancer drug. Bioorg Chem. 88, 102925. 10.1016/j.bioorg.2019.102925 31003078

[B42] Goodwin J. M. Dowdle W. E. DeJesus R. Wang Z. Bergman P. Kobylarz M. (2017). Autophagy-independent lysosomal targeting regulated by ULK1/2-fip200 and ATG9. Cell Rep. 20, 2341–2356. 10.1016/j.celrep.2017.08.034 28877469 PMC5699710

[B43] Grotzke J. E. Kozik P. Morel J. D. Impens F. Pietrosemoli N. Cresswell P. (2017). Sec61 blockade by mycolactone inhibits antigen cross-presentation independently of endosome-to-cytosol export. Proc. Natl. Acad. Sci. U. S. A. 114, E5910–e5919. 10.1073/pnas.1705242114 28679634 PMC5530691

[B44] Gubas A. Dikic I. (2022). ER remodeling via ER-phagy. Mol. Cell 82, 1492–1500. 10.1016/j.molcel.2022.02.018 35452617 PMC9098120

[B45] Haeri M. Knox B. E. (2012). Endoplasmic reticulum stress and unfolded protein response pathways: potential for treating age-related retinal degeneration. J. Ophthalmic Vis. Res. 7, 45–59. 22737387 PMC3381108

[B46] He Y. Li W. Zhang J. Yang Y. Qian Y. Zhou D. (2021). The curcumin analog EF24 is highly active against chemotherapy- resistant melanoma cells. Curr. Cancer Drug Targets 21, 608–618. 10.2174/1568009621666210303092921 33655859

[B47] Healy S. J. Gorman A. M. Mousavi-Shafaei P. Gupta S. Samali A. (2009). Targeting the endoplasmic reticulum-stress response as an anticancer strategy. Eur. J. Pharmacol. 625, 234–246. 10.1016/j.ejphar.2009.06.064 19835867

[B48] Hetz C. Zhang K. Kaufman R. J. (2020). Mechanisms, regulation and functions of the unfolded protein response. Nat. Rev. Mol. Cell Biol. 21, 421–438. 10.1038/s41580-020-0250-z 32457508 PMC8867924

[B49] Heyza J. R. Arora S. Zhang H. Conner K. L. Lei W. Floyd A. M. (2018). Targeting the DNA repair endonuclease ERCC1-XPF with green tea polyphenol epigallocatechin-3-gallate (EGCG) and its prodrug to enhance cisplatin efficacy in human cancer cells. Nutrients 10, 1644. 10.3390/nu10111644 30400270 PMC6267282

[B50] Hiraishi H. Oatman J. Haller S. L. Blunk L. McGivern B. Morris J. (2014). Essential role of eIF5-mimic protein in animal development is linked to control of ATF4 expression. Nucleic Acids Res. 42, 10321–10330. 10.1093/nar/gku670 25147208 PMC4176352

[B51] Hosomi A. Tanabe K. Hirayama H. Kim I. Rao H. Suzuki T. (2010). Identification of an Htm1 (EDEM)-dependent, mns1-independent endoplasmic reticulum-associated degradation (ERAD) pathway in *Saccharomyces cerevisiae*: application of a novel assay for glycoprotein ERAD. J. Biol. Chem. 285, 24324–24334. 10.1074/jbc.M109.095919 20511219 PMC2915668

[B52] Hotamisligil G. S. Davis R. J. (2016). Cell signaling and stress responses. Cold Spring Harb. Perspect. Biol. 8, a006072. 10.1101/cshperspect.a006072 27698029 PMC5046695

[B53] Huang F. Wang B. R. Wang Y. G. (2018). Role of autophagy in tumorigenesis, metastasis, targeted therapy and drug resistance of hepatocellular carcinoma. World J. Gastroenterol. 24, 4643–4651. 10.3748/wjg.v24.i41.4643 30416312 PMC6224467

[B54] Hübner C. A. Dikic I. (2020). ER-phagy and human diseases. Cell Death Differ. 27, 833–842. 10.1038/s41418-019-0444-0 31659280 PMC7206075

[B55] Hussein D. Alsereihi R. Salwati A. A. A. Algehani R. Alhowity A. Al-Hejin A. M. (2022). The anterior gradient homologue 2 (AGR2) co-localises with the glucose-regulated protein 78 (GRP78) in cancer stem cells, and is critical for the survival and drug resistance of recurrent glioblastoma: *in situ* and *in vitro* analyses. Cancer Cell Int. 22, 387. 10.1186/s12935-022-02814-5 36482387 PMC9730595

[B56] Ibrahim I. M. Abdelmalek D. H. Elfiky A. A. (2019). GRP78: a cell's response to stress. Life Sci. 226, 156–163. 10.1016/j.lfs.2019.04.022 30978349 PMC7094232

[B57] Ishaq M. Ojha R. Sharma A. P. Singh S. K. (2020). Autophagy in cancer: recent advances and future directions. Semin. Cancer Biol. 66, 171–181. 10.1016/j.semcancer.2020.03.010 32201367

[B58] Jamali B. Nakhjavani M. Hosseinzadeh L. Amidi S. Nikounezhad N. H Shirazi F. (2015). Intracellular GSH alterations and its relationship to level of resistance following exposure to cisplatin in cancer cells. Iran. J. Pharm. Res. 14, 513–519. 25901159 PMC4403068

[B59] Jiang Q. Li F. Shi K. Wu P. An J. Yang Y. (2014). Involvement of p38 in signal switching from autophagy to apoptosis via the PERK/eIF2α/ATF4 axis in selenite-treated NB4 cells. Cell Death Dis. 5, e1270. 10.1038/cddis.2014.200 24874742 PMC4047911

[B60] Kang J. A. Jeon Y. J. (2021). How is the fidelity of proteins ensured in terms of both quality and quantity at the endoplasmic reticulum? Mechanistic insights into E3 ubiquitin ligases. Int. J. Mol. Sci. 22, 2078. 10.3390/ijms22042078 33669844 PMC7923238

[B61] Kim H. Bhattacharya A. Qi L. (2015). Endoplasmic reticulum quality control in cancer: friend or foe. Semin. Cancer Biol. 33, 25–33. 10.1016/j.semcancer.2015.02.003 25794824 PMC4523434

[B62] Kim I. Xu W. Reed J. C. (2008). Cell death and endoplasmic reticulum stress: disease relevance and therapeutic opportunities. Nat. Rev. Drug Discov. 7, 1013–1030. 10.1038/nrd2755 19043451

[B63] Kim P. Scott M. R. Meador-Woodruff J. H. (2018). Abnormal expression of ER quality control and ER associated degradation proteins in the dorsolateral prefrontal cortex in schizophrenia. Schizophr. Res. 197, 484–491. 10.1016/j.schres.2018.02.010 29496332 PMC6109614

[B64] Kryczka J. Kryczka J. Czarnecka-Chrebelska K. H. Brzeziańska-Lasota E. (2021). Molecular mechanisms of chemoresistance induced by cisplatin in NSCLC cancer therapy. Int. J. Mol. Sci. 22, 8885. 10.3390/ijms22168885 34445588 PMC8396273

[B65] Lau A. Villeneuve N. F. Sun Z. Wong P. K. Zhang D. D. (2008). Dual roles of Nrf2 in cancer. Pharmacol. Res. 58, 262–270. 10.1016/j.phrs.2008.09.003 18838122 PMC2652397

[B66] Lebeaupin C. Vallée D. Hazari Y. Hetz C. Chevet E. Bailly-Maitre B. (2018). Endoplasmic reticulum stress signalling and the pathogenesis of non-alcoholic fatty liver disease. J. Hepatol. 69, 927–947. 10.1016/j.jhep.2018.06.008 29940269

[B67] Lemberg M. K. Strisovsky K. (2021). Maintenance of organellar protein homeostasis by ER-associated degradation and related mechanisms. Mol. Cell 81, 2507–2519. 10.1016/j.molcel.2021.05.004 34107306

[B68] Lewis-Wambi J. S. Jordan V. C. (2009). Estrogen regulation of apoptosis: how can one hormone stimulate and inhibit? Breast Cancer Res. 11, 206. 10.1186/bcr2255 19519952 PMC2716493

[B69] Li F. Zheng Z. Chen W. Li D. Zhang H. Zhu Y. (2023). Regulation of cisplatin resistance in bladder cancer by epigenetic mechanisms. Drug Resist Updat 68, 100938. 10.1016/j.drup.2023.100938 36774746

[B70] Lin Y. Jiang M. Chen W. Zhao T. Wei Y. (2019). Cancer and ER stress: mutual crosstalk between autophagy, oxidative stress and inflammatory response. Biomed. Pharmacother. 118, 109249. 10.1016/j.biopha.2019.109249 31351428

[B71] Linder S. Shoshan M. C. (2005). Lysosomes and endoplasmic reticulum: targets for improved, selective anticancer therapy. Drug Resist Updat 8, 199–204. 10.1016/j.drup.2005.06.004 16055370

[B72] Liu C. Yan D. Y. Wang C. Ma Z. Deng Y. Liu W. (2020). IRE1 signaling pathway mediates protective autophagic response against manganese-induced neuronal apoptosis *in vivo* and *in vitro* . Sci. Total Environ. 712, 136480. 10.1016/j.scitotenv.2019.136480 31931206

[B73] Liu X. Su K. Sun X. Jiang Y. Wang L. Hu C. (2021). Sec62 promotes stemness and chemoresistance of human colorectal cancer through activating Wnt/β-catenin pathway. J. Exp. Clin. Cancer Res. 40, 132. 10.1186/s13046-021-01934-6 33858476 PMC8051072

[B74] Lugones Y. Loren P. Salazar L. A. (2022). Cisplatin resistance: genetic and epigenetic factors involved. Biomolecules 12, 1365. 10.3390/biom12101365 36291573 PMC9599500

[B75] Lukanović D. Herzog M. Kobal B. Černe K. (2020). The contribution of copper efflux transporters ATP7A and ATP7B to chemoresistance and personalized medicine in ovarian cancer. Biomed. Pharmacother. 129, 110401. 10.1016/j.biopha.2020.110401 32570116

[B76] Luo Y. Xiang W. Liu Z. Yao L. Tang L. Tan W. (2022). Functional role of the SLC7A11-AS1/xCT axis in the development of gastric cancer cisplatin-resistance by a GSH-dependent mechanism. Free Radic. Biol. Med. 184, 53–65. 10.1016/j.freeradbiomed.2022.03.026 35367340

[B77] Ma M. Lin X. H. Liu H. H. Zhang R. Chen R. X. (2020). Suppression of DRP1-mediated mitophagy increases the apoptosis of hepatocellular carcinoma cells in the setting of chemotherapy. Oncol. Rep. 43, 1010–1018. 10.3892/or.2020.7476 32020220

[B78] Mandic A. Hansson J. Linder S. Shoshan M. C. (2003). Cisplatin induces endoplasmic reticulum stress and nucleus-independent apoptotic signaling. J. Biol. Chem. 278, 9100–9106. 10.1074/jbc.M210284200 12509415

[B79] Maurel M. Chevet E. Tavernier J. Gerlo S. (2014). Getting RIDD of RNA: IRE1 in cell fate regulation. Trends Biochem. Sci. 39, 245–254. 10.1016/j.tibs.2014.02.008 24657016

[B80] Ménager J. Ebstein F. Oger R. Hulin P. Nedellec S. Duverger E. (2014). Cross-presentation of synthetic long peptides by human dendritic cells: a process dependent on ERAD component p97/VCP but Not sec61 and/or Derlin-1. PLoS One 9, e89897. 10.1371/journal.pone.0089897 24587108 PMC3937416

[B81] Morris J. A. Dorner A. J. Edwards C. A. Hendershot L. M. Kaufman R. J. (1997). Immunoglobulin binding protein (BiP) function is required to protect cells from endoplasmic reticulum stress but is not required for the secretion of selective proteins. J. Biol. Chem. 272, 4327–4334. 10.1074/jbc.272.7.4327 9020152

[B82] Mosca L. Pagano M. Ilisso C. P. Cave D. D. Desiderio V. Mele L. (2019). AdoMet triggers apoptosis in head and neck squamous cancer by inducing ER stress and potentiates cell sensitivity to cisplatin. J. Cell Physiol. 234, 13277–13291. 10.1002/jcp.28000 30575033 PMC13483283

[B83] Nakagawa H. Ohira M. Hayashi S. Abe S. Saito S. Nagahori N. (2008). Alterations in the glycoform of cisplatin-resistant human carcinoma cells are caused by defects in the endoplasmic reticulum-associated degradation system. Cancer Lett. 270, 295–301. 10.1016/j.canlet.2008.05.019 18573595

[B84] Nakatsukasa K. Brodsky J. L. (2008). The recognition and retrotranslocation of misfolded proteins from the endoplasmic reticulum. Traffic 9, 861–870. 10.1111/j.1600-0854.2008.00729.x 18315532 PMC2754126

[B85] Nawrocki S. T. Carew J. S. Pino M. S. Highshaw R. A. Dunner K. Huang P. (2005). Bortezomib sensitizes pancreatic cancer cells to endoplasmic reticulum stress-mediated apoptosis. Cancer Res. 65, 11658–11666. 10.1158/0008-5472.Can-05-2370 16357177

[B86] Nowis D. McConnell E. Wójcik C. (2006). Destabilization of the VCP-Ufd1-Npl4 complex is associated with decreased levels of ERAD substrates. Exp. Cell Res. 312, 2921–2932. 10.1016/j.yexcr.2006.05.013 16822501

[B87] Oakes S. A. Papa F. R. (2015). The role of endoplasmic reticulum stress in human pathology. Annu. Rev. Pathol. 10, 173–194. 10.1146/annurev-pathol-012513-104649 25387057 PMC5568783

[B88] Oizumi S. Isobe H. Ogura S. Ishida T. Yamazaki K. Nishimura M. (2002). Topoisomerase inhibitor-induced apoptosis accompanied by down-regulation of Bcl-2 in human lung cancer cells. Anticancer Res. 22, 4029–4037. 12553028

[B89] Pi L. Li X. Song Q. Shen Y. Lu X. Di B. (2014). Knockdown of glucose-regulated protein 78 abrogates chemoresistance of hypopharyngeal carcinoma cells to cisplatin induced by unfolded protein in response to severe hypoxia. Oncol. Lett. 7, 685–692. 10.3892/ol.2013.1753 24527073 PMC3919852

[B90] Piirainen M. A. Frey A. D. (2022). The impact of glycoengineering on the endoplasmic reticulum quality control system in yeasts. Front. Mol. Biosci. 9, 910709. 10.3389/fmolb.2022.910709 35720120 PMC9201249

[B91] Preetha Rani M. R. Salin Raj P. Nair A. Ranjith S. Rajankutty K. Raghu K. G. (2022). *In vitro* and *in vivo* studies reveal the beneficial effects of chlorogenic acid against ER stress mediated ER-phagy and associated apoptosis in the heart of diabetic rat. Chem. Biol. Interact. 351, 109755. 10.1016/j.cbi.2021.109755 34801538

[B92] Qi L. Tsai B. Arvan P. (2017). New insights into the physiological role of endoplasmic reticulum-associated degradation. Trends Cell Biol. 27, 430–440. 10.1016/j.tcb.2016.12.002 28131647 PMC5440201

[B93] Qiu L. Zheng X. Jaishankar D. Green R. Fang D. Nadig S. (2023). Beyond UPR: cell-specific roles of ER stress sensor IRE1α in kidney ischemic injury and transplant rejection. Kidney Int. 104, 463–469. 10.1016/j.kint.2023.06.016 37391039 PMC10519186

[B94] Ramos-Gomez M. Dolan P. M. Itoh K. Yamamoto M. Kensler T. W. (2003). Interactive effects of nrf2 genotype and oltipraz on benzo[a]pyrene-DNA adducts and tumor yield in mice. Carcinogenesis 24, 461–467. 10.1093/carcin/24.3.461 12663505

[B95] Ray Chaudhuri A. Callen E. Ding X. Gogola E. Duarte A. A. Lee J. E. (2016). Replication fork stability confers chemoresistance in BRCA-deficient cells. Nature 535, 382–387. 10.1038/nature18325 27443740 PMC4959813

[B96] Reggiori F. Molinari M. (2022). ER-phagy: mechanisms, regulation, and diseases connected to the lysosomal clearance of the endoplasmic reticulum. Physiol. Rev. 102, 1393–1448. 10.1152/physrev.00038.2021 35188422 PMC9126229

[B97] Ren J.-H. He W.-S. Nong L. Zhu Q.-Y. Hu K. Zhang R.-G. (2010). Acquired cisplatin resistance in human lung adenocarcinoma cells is associated with enhanced autophagy. Cancer Biother Radiopharm. 25, 75–80. 10.1089/cbr.2009.0701 20187799

[B98] Ricciardi C. A. Gnudi L. (2020). The endoplasmic reticulum stress and the unfolded protein response in kidney disease: implications for vascular growth factors. J. Cell Mol. Med. 24, 12910–12919. 10.1111/jcmm.15999 33067928 PMC7701511

[B99] Riemer J. Hansen H. G. Appenzeller-Herzog C. Johansson L. Ellgaard L. (2011). Identification of the PDI-family member ERp90 as an interaction partner of ERFAD. PLoS One 6, e17037. 10.1371/journal.pone.0017037 21359175 PMC3040216

[B100] Rodriguez-Antona C. Ingelman-Sundberg M. (2006). Cytochrome P450 pharmacogenetics and cancer. Oncogene 25, 1679–1691. 10.1038/sj.onc.1209377 16550168

[B101] Romani A. M. P. (2022). Cisplatin in cancer treatment. Biochem. Pharmacol. 206, 115323. 10.1016/j.bcp.2022.115323 36368406

[B102] Römisch K. (2005). Endoplasmic reticulum-associated degradation. Annu. Rev. Cell Dev. Biol. 21, 435–456. 10.1146/annurev.cellbio.21.012704.133250 16212502

[B103] Rotblat B. Melino G. Knight R. A. (2012). NRF2 and p53: januses in cancer? Oncotarget 3, 1272–1283. 10.18632/oncotarget.754 23174755 PMC3717791

[B104] Rozpedek W. Pytel D. Mucha B. Leszczynska H. Diehl J. A. Majsterek I. (2016). The role of the PERK/eIF2α/ATF4/CHOP signaling pathway in tumor progression during endoplasmic reticulum stress. Curr. Mol. Med. 16, 533–544. 10.2174/1566524016666160523143937 27211800 PMC5008685

[B105] Ruddock L. W. Molinari M. (2006). N-glycan processing in ER quality control. J. Cell Sci. 119, 4373–4380. 10.1242/jcs.03225 17074831

[B106] Salaroglio I. C. Panada E. Moiso E. Buondonno I. Provero P. Rubinstein M. (2017). PERK induces resistance to cell death elicited by endoplasmic reticulum stress and chemotherapy. Mol. Cancer 16, 91. 10.1186/s12943-017-0657-0 28499449 PMC5427528

[B107] Samimi G. Fink D. Varki N. M. Husain A. Hoskins W. J. Alberts D. S. (2000). Analysis of MLH1 and MSH2 expression in ovarian cancer before and after platinum drug-based chemotherapy. Clin. Cancer Res. 6, 1415–1421. 10778972

[B108] Sasset L. Petris G. Cesaratto F. Burrone O. R. (2015). The VCP/p97 and YOD1 proteins have different substrate-dependent activities in endoplasmic reticulum-associated degradation (ERAD). J. Biol. Chem. 290, 28175–28188. 10.1074/jbc.M115.656660 26463207 PMC4653676

[B109] Senft D. Ronai Z. A. (2015). UPR, autophagy, and mitochondria crosstalk underlies the ER stress response. Trends Biochem. Sci. 40, 141–148. 10.1016/j.tibs.2015.01.002 25656104 PMC4340752

[B110] Singh A. Venkannagari S. Oh K. H. Zhang Y. Q. Rohde J. M. Liu L. (2016). Small molecule inhibitor of NRF2 selectively intervenes therapeutic resistance in KEAP1-deficient NSCLC tumors. ACS Chem. Biol. 11, 3214–3225. 10.1021/acschembio.6b00651 27552339 PMC5367156

[B111] Słomińska-Wojewódzka M. Sandvig K. (2015). The role of lectin-carbohydrate interactions in the regulation of ER-associated protein degradation. Molecules 20, 9816–9846. 10.3390/molecules20069816 26023941 PMC6272441

[B112] Smith A. G. Macleod K. F. (2019). Autophagy, cancer stem cells and drug resistance. J. Pathol. 247, 708–718. 10.1002/path.5222 30570140 PMC6668344

[B113] Song S. Tan J. Miao Y. Zhang Q. (2018). Crosstalk of ER stress-mediated autophagy and ER-phagy: involvement of UPR and the core autophagy machinery. J. Cell Physiol. 233, 3867–3874. 10.1002/jcp.26137 28777470

[B114] Sui X. Chen R. Wang Z. Huang Z. Kong N. Zhang M. (2013). Autophagy and chemotherapy resistance: a promising therapeutic target for cancer treatment. Cell Death Dis. 4, e838. 10.1038/cddis.2013.350 24113172 PMC3824660

[B115] Sun Y. Qiao Y. Liu Y. Zhou J. Wang X. Zheng H. (2021). ent-Kaurane diterpenoids induce apoptosis and ferroptosis through targeting redox resetting to overcome cisplatin resistance. Redox Biol. 43, 101977. 10.1016/j.redox.2021.101977 33905957 PMC8099784

[B116] Tang B. Li Q. Zhao X.-H. Wang H.-G. Li N. Fang Y. (2015). Shiga toxins induce autophagic cell death in intestinal epithelial cells via the endoplasmic reticulum stress pathway. Autophagy 11, 344–354. 10.1080/15548627.2015.1023682 25831014 PMC4502731

[B117] Tang H. Y. Huang C. H. Zhuang Y. H. Christianson J. C. Chen X. (2014). EDEM2 and OS-9 are required for ER-associated degradation of non-glycosylated sonic hedgehog. PLoS One 9, e92164. 10.1371/journal.pone.0092164 24910992 PMC4049591

[B118] Tang Z. Du W. Xu F. Sun X. Chen W. Cui J. (2022). Icariside II enhances cisplatin-induced apoptosis by promoting endoplasmic reticulum stress signalling in non-small cell lung cancer cells. Int. J. Biol. Sci. 18, 2060–2074. 10.7150/ijbs.66630 35342361 PMC8935239

[B119] Ting S. Mairinger F. D. Hager T. Welter S. Eberhardt W. E. Wohlschlaeger J. (2013). ERCC1, MLH1, MSH2, MSH6, and βIII-tubulin: resistance proteins associated with response and outcome to platinum-based chemotherapy in malignant pleural mesothelioma. Clin. Lung Cancer 14, 558–567. 10.1016/j.cllc.2013.04.013 23810210

[B120] Urra H. Dufey E. Avril T. Chevet E. Hetz C. (2016). Endoplasmic reticulum stress and the hallmarks of cancer. Trends Cancer 2, 252–262. 10.1016/j.trecan.2016.03.007 28741511

[B121] van der Goot A. T. Pearce M. M. P. Leto D. E. Shaler T. A. Kopito R. R. (2018). Redundant and antagonistic roles of XTP3B and OS9 in decoding glycan and non-glycan degrons in ER-associated degradation. Mol. Cell 70, 516–530. 10.1016/j.molcel.2018.03.026 29706535 PMC5935522

[B122] Vianello C. Cocetta V. Catanzaro D. Dorn G. W. 2nd De Milito A. Rizzolio F. (2022). Cisplatin resistance can be curtailed by blunting Bnip3-mediated mitochondrial autophagy. Cell Death Dis. 13, 398. 10.1038/s41419-022-04741-9 35459212 PMC9033831

[B123] Walter P. Ron D. (2011). The unfolded protein response: from stress pathway to homeostatic regulation. Science 334, 1081–1086. 10.1126/science.1209038 22116877

[B124] Wang J. Kang R. Huang H. Xi X. Wang B. Wang J. (2014). Hepatitis C virus core protein activates autophagy through EIF2AK3 and ATF6 UPR pathway-mediated MAP1LC3B and ATG12 expression. Autophagy 10, 766–784. 10.4161/auto.27954 24589849 PMC5119055

[B125] Wang M. Kaufman R. J. (2014). The impact of the endoplasmic reticulum protein-folding environment on cancer development. Nat. Rev. Cancer 14, 581–597. 10.1038/nrc3800 25145482

[B126] Wang M. Kaufman R. J. (2016). Protein misfolding in the endoplasmic reticulum as a conduit to human disease. Nature 529, 326–335. 10.1038/nature17041 26791723

[B127] Xu D. Liang S. Q. Yang H. Lüthi U. Riether C. Berezowska S. (2018). Increased sensitivity to apoptosis upon endoplasmic reticulum stress-induced activation of the unfolded protein response in chemotherapy-resistant malignant pleural mesothelioma. Br. J. Cancer 119, 65–75. 10.1038/s41416-018-0145-3 29921948 PMC6035279

[B128] Xu D. Liu Z. Liang M. X. Fei Y. J. Zhang W. Wu Y. (2022). Endoplasmic reticulum stress targeted therapy for breast cancer. Cell Commun. Signal 20, 174. 10.1186/s12964-022-00964-7 36345017 PMC9639265

[B129] Xu J. Gewirtz D. A. (2022). Is autophagy always a barrier to cisplatin therapy? Biomolecules 12, 463. 10.3390/biom12030463 35327655 PMC8946631

[B130] Xu J. Patel N. H. Gewirtz D. A. (2020a). Triangular relationship between p53, autophagy, and chemotherapy resistance. Int. J. Mol. Sci. 21, 8991. 10.3390/ijms21238991 33256191 PMC7730978

[B131] Xu Z. Han X. Ou D. Liu T. Li Z. Jiang G. (2020b). Targeting PI3K/AKT/mTOR-mediated autophagy for tumor therapy. Appl. Microbiol. Biotechnol. 104, 575–587. 10.1007/s00253-019-10257-8 31832711

[B132] Yan M. Ni J. Song D. Ding M. Huang J. (2015). Activation of unfolded protein response protects osteosarcoma cells from cisplatin-induced apoptosis through NF-κB pathway. Int. J. Clin. Exp. Pathol. 8, 10204–10215. 26617729 PMC4637544

[B133] Yu F. Megyesi J. Price P. M. (2008). Cytoplasmic initiation of cisplatin cytotoxicity. Am. J. Physiol. Ren. Physiol. 295, F44–F52. 10.1152/ajprenal.00593.2007 PMC2494516 18400869

[B134] Yu F. Megyesi J. Safirstein R. L. Price P. M. (2007). Involvement of the CDK2-E2F1 pathway in cisplatin cytotoxicity *in vitro* and *in vivo* . Am. J. Physiol. Ren. Physiol. 293, F52–F59. 10.1152/ajprenal.00119.2007 17459956

[B135] Yu G. Klionsky D. J. (2022). Life and death decisions-the many faces of autophagy in cell survival and cell death. Biomolecules 12, 866. 10.3390/biom12070866 35883421 PMC9313301

[B136] Zamame Ramirez J. A. Romagnoli G. G. Kaneno R. (2021). Inhibiting autophagy to prevent drug resistance and improve anti-tumor therapy. Life Sci. 265, 118745. 10.1016/j.lfs.2020.118745 33186569

[B137] Zattas D. Hochstrasser M. (2015). Ubiquitin-dependent protein degradation at the yeast endoplasmic reticulum and nuclear envelope. Crit. Rev. Biochem. Mol. Biol. 50, 1–17. 10.3109/10409238.2014.959889 25231236 PMC4359062

[B138] Zhang B. Liu L. (2021). Autophagy is a double-edged sword in the therapy of colorectal cancer. Oncol. Lett. 21, 378. 10.3892/ol.2021.12639 33777202 PMC7988732

[B139] Zhang J. Wu J. Liu L. Li J. (2020). The crucial role of demannosylating asparagine-linked glycans in ERADicating misfolded glycoproteins in the endoplasmic reticulum. Front. Plant Sci. 11, 625033. 10.3389/fpls.2020.625033 33510762 PMC7835635

[B140] Zhang L. Littlejohn J. E. Cui Y. Cao X. Peddaboina C. Smythe W. R. (2010). Characterization of bortezomib-adapted I-45 mesothelioma cells. Mol. Cancer 9, 110. 10.1186/1476-4598-9-110 20482802 PMC2882347

[B141] Zhang M. Duan X. Wang L. Wen J. Fang P. (2023). Deregulation of HSF1-mediated endoplasmic reticulum unfolded protein response promotes cisplatin resistance in lung cancer cells. Febs J. 290, 2706–2720. 10.1111/febs.16709 36536996

[B142] Zhang S. X. Sanders E. Fliesler S. J. Wang J. J. (2014). Endoplasmic reticulum stress and the unfolded protein responses in retinal degeneration. Exp. Eye Res. 125, 30–40. 10.1016/j.exer.2014.04.015 24792589 PMC4122592

[B143] Zhou Z. Torres M. Sha H. Halbrook C. J. Van den Bergh F. Reinert R. B. (2020). Endoplasmic reticulum-associated degradation regulates mitochondrial dynamics in brown adipocytes. Science 368, 54–60. 10.1126/science.aay2494 32193362 PMC7409365

[B144] Zhu C. Xie Y. Li Q. Zhang Z. Chen J. Zhang K. (2023). CPSF6-mediated XBP1 3'UTR shortening attenuates cisplatin-induced ER stress and elevates chemo-resistance in lung adenocarcinoma. Drug Resist Updat 68, 100933. 10.1016/j.drup.2023.100933 36821972

[B145] Zielke S. Kardo S. Zein L. Mari M. Covarrubias-Pinto A. Kinzler M. N. (2021). ATF4 links ER stress with reticulophagy in glioblastoma cells. Autophagy 17, 1–17. 10.1080/15548627.2020.1827780 33111629 PMC8496713

